# Pesticide Residues in Pome Fruits: Occurrence, Quality Profiling, and Advanced Dietary Risk Characterisation

**DOI:** 10.3390/molecules31122132

**Published:** 2026-06-17

**Authors:** Nimo Hussein Yussuf, Tuba Buyuksirit-Bedir, Cagla Kayisoglu, Eylem Odabas, Fatma Oznur Afacan, Ozgur Golge, Tamara Lazarević-Pašti, Bulent Kabak

**Affiliations:** 1 Department of Nutrition and Food Science, Edna Adan University, Iftin Road, Hargeisa 19030, Somaliland, Somalia; qadanbudhle@gmail.com; 2 Department of Food Engineering, Faculty of Engineering and Natural Sciences, Hitit University, Corum 19030, Türkiye; tubabuyuksirit@hitit.edu.tr (T.B.-B.); eylemodabas19@gmail.com (E.O.); 3 Scientific Technical Application and Research Center, Hitit University, Corum 19030, Türkiye; caglakayisoglu@hitit.edu.tr; 4 Department of Nutrition and Dietetics, Hamidiye Faculty of Health Sciences, University of Health Sciences, Istanbul 34668, Türkiye; ftmznr@gmail.com; 5 Department of Gastronomy and Culinary Arts, Faculty of Tourism, Alanya Alaaddin Keykubat University, Alanya 07425, Türkiye; ozgur.golge@alanya.edu.tr; 6 Department of Physical Chemistry, VINČA Institute of Nuclear Sciences—National Institute of the Republic of Serbia, University of Belgrade, 11000 Belgrade, Serbia

**Keywords:** apple, pear, quince, pesticides, risk ranking, hazard index, Monte Carlo simulation, QuEChERS, LC-MS/MS

## Abstract

The occurrence of pesticide residues in pome fruits and their implications for consumer health remain critical concerns in food safety. In this study, 222 pesticide residues were analysed in 155 samples of apples, pears, and quinces collected from Türkiye between October 2025 and March 2026 using liquid chromatography–tandem mass spectrometry (LC-MS/MS). Residues were detected in 76.4% of apples, 86% of pears, and 30% of quinces, with frequent multi-residue patterns and notable occurrences of non-approved compounds. Pear samples exhibited the highest contamination levels, with maximum residue level (MRL) exceedance rates reaching 30%, compared to 14.5% in apples and 2% in quinces. Quality assessment based on the index of quality for residues (*IqR*) indicated that 96% of quince samples were classified as excellent or good, demonstrating the most favourable profile among the evaluated commodities. Risk ranking analysis further indicated that acetamiprid was the only high-risk pesticide in apples, whereas residues in pears were predominantly medium risk, and all detected compounds in quinces fell within the low-risk category. Deterministic risk assessment indicated that chronic exposure remained well below levels of concern for both adults and children. Under combined pome fruit consumption, acetamiprid and spirodiclofen were identified as the main contributors to chronic hazard index (*HIc*), accounting for 33% and 13% of *HIc*, respectively. However, acute exposure exceeded the safety threshold (*HQa* > 1) in children for acetamiprid in both apples and pears. Probabilistic modelling confirmed right-skewed exposure distributions and highlighted increased risk under cumulative consumption scenarios.

## 1. Introduction

The intensive use of pesticides has become an indispensable component of modern agricultural systems aimed at sustaining global food production and ensuring crop quality. While these compounds play a critical role in protecting crops against pests and diseases and minimizing yield losses, their widespread, often uncontrolled use has raised concerns about environmental sustainability and human health. With continued global population growth, agricultural intensification has become unavoidable, further reinforcing reliance on pesticide-based interventions [1]. It has been estimated that nearly one-third of global agricultural production depends directly on pesticide use [2]. However, the persistence and bioaccumulation of these compounds in environmental matrices, including soil, water, and air, have been shown to adversely affect not only target organisms but also a wide range of non-target species across terrestrial and aquatic ecosystems [3,4,5].

In addition to environmental contamination, dietary exposure represents one of the most significant pathways through which humans are exposed to pesticide residues. The presence of these residues in food has been associated with a variety of adverse health outcomes, including carcinogenicity, neurotoxicity, reproductive disorders, and genotoxic effects, supported by both mechanistic and epidemiological evidence [4,5,6]. Vulnerable populations, particularly infants and children, are at higher risk due to their ongoing physiological development and immature metabolic systems [6].

Given these concerns, regulatory authorities have established strict control measures to limit pesticide residues in food commodities. Maximum residue levels (MRLs) have been defined to ensure that pesticide concentrations remain within levels considered safe for human consumption [7]. Alongside regulatory limits, sustainable agricultural strategies, such as Integrated Pest Management (IPM), have been promoted to reduce dependency on chemical pesticides [1]. Despite these regulatory and technological efforts, pesticide residues remain among the most frequently reported hazards in global food safety monitoring systems. Data from the Rapid Alert System for Food and Feed (RASFF) indicate that pesticides were the second-most-reported hazard category between January 2020 and March 2026, with a total of 5877 notifications. Fruits and vegetables were identified as the most affected commodity group, accounting for 3359 notifications [8].

Pome fruits, including apple (*Malus domestica*), pear (*Pyrus* L.), and quince (*Cydonia oblonga* Mill.), are among the most widely consumed fruits worldwide and represent a significant component of both human nutrition and international agricultural trade. These fruits are consumed both fresh and in various processed forms, including juices, purees, jams, dried products, and fermented products such as vinegar and cider. Their nutritional value is attributed to their high dietary fiber content, particularly pectin, as well as vitamins, organic acids, and bioactive compounds with antioxidant and anti-inflammatory properties [9,10]. The World Health Organization has widely promoted the consumption of fruits and vegetables as part of global public health strategies due to their protective effects against chronic diseases [11,12].

However, pome fruits are highly susceptible to fungal infections and pest infestations throughout production, storage, and distribution, which can compromise both product quality and marketability. These commodities are particularly vulnerable to pathogens such as *Penicillium expansum*, *Botrytis cinerea*, *Alternaria* spp., and *Rhizopus stolonifer*, as well as various insect pests. Therefore, pesticide applications are widely employed across the production chain to ensure yield stability and post-harvest quality [13].

Because pome fruits are commonly consumed unpeeled or with minimal processing, surface-associated pesticide residues are less effectively removed, which may contribute to higher dietary exposure compared with fruits that are routinely peeled before consumption [14]. It raises significant concerns regarding dietary exposure and the potential exceedance of established MRLs. Although numerous studies have investigated pesticide residues in apples [15,16,17,18,19,20,21,22], relatively fewer studies have focused on pears [23,24,25,26,27,28], and even fewer on quinces [29,30,31]. More importantly, studies jointly evaluating these commodities remain limited. To the best of current knowledge, no comprehensive study has simultaneously assessed pesticide residues in apple, pear, and quince samples and evaluated their cumulative dietary exposure using both deterministic and probabilistic approaches.

The present study aimed to determine multi-residue pesticide levels in pome fruits, including apple (*n* = 55), pear (*n* = 50), and quince (*n* = 50), using liquid chromatography–tandem mass spectrometry (LC-MS/MS), and to assess their compliance with European Union (EU) MRLs. In addition, quality evaluation based on the Index of Quality for Residues (*IqR*) and risk ranking were performed. Chronic and acute dietary exposure assessments were conducted for both adults and children based on individual and combined consumption scenarios using both deterministic and probabilistic approaches.

## 2. Results and Discussion

### 2.1. Occurrence and Distribution of Pesticide Residues in Pome Fruits

A total of 155 pome fruit samples, comprising apple (*n* = 55), pear (*n* = 50), and quince (*n* = 50), collected from Türkiye, were analysed for 222 pesticide residues using LC-MS/MS. The distribution and concentrations of detected pesticides are presented in Table 1.

**Figure 1:**
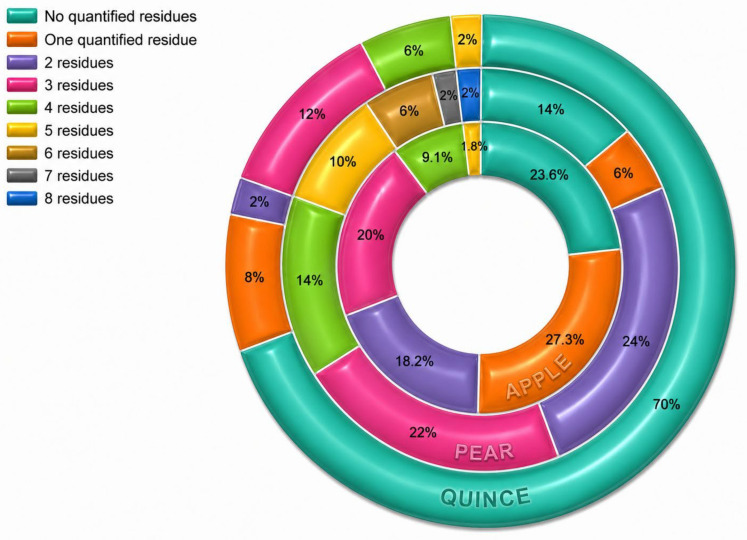
Distribution of residue occurrence patterns in pome fruits, illustrating the number of pesticide residues detected per sample and the prevalence of multi-residue profiles. The inner, middle, and outer rings correspond to apple, pear, and quince samples, respectively.

Pesticide residues were detected in 42 out of 55 apple samples (76.4%), whereas 13 samples were free of detectable residues. Among contaminated samples, 8 (14.5%) exceeded the EU MRLs. Single residues were identified in 15 samples, while 27 samples contained multiple residues, including up to five different compounds. In pear samples, residues were detected in 43 of 50 (86%), indicating a higher contamination frequency than in apples. Notably, 15 samples (30%) contained at least one pesticide residue exceeding the EU MRLs. Multi-residue occurrence was dominant, with up to eight different pesticides detected in a single sample, reflecting a more intensive chemical input in pear production. In contrast, quince samples exhibited substantially lower contamination levels, with detectable residues identified in only 15 out of 50 samples (30%), while only one sample (2%) exceeded the EU MRLs. Although multi-residue occurrence (22%) was observed, the number of detected compounds remained comparatively limited. The distribution of pesticide residue occurrence patterns in pome fruit samples is presented in Figure 1.

In total, 15, 18, and 10 different pesticide residues were identified in apple, pear, and quince samples, respectively. The presence of non-approved pesticides, particularly in apples (*n* = 4), pears (*n* = 4), and quinces (*n* = 2), highlights differences in regulatory authorisation status between the EU and Türkiye and suggests variability in pesticide management practices. Notably, thiophanate-methyl, a systemic fungicide previously used for the control of fungal diseases, was detected across all three fruit matrices. This compound is no longer approved for use in the EU, with authorisations withdrawn by April 2021 and a maximum grace period ending in October 2021 [7]. However, the compounds classified as non-approved in the present study were authorised for use in Türkiye during the sampling period. Therefore, their detection should not be interpreted as evidence of illegal application but rather as an indication of differences between national and EU regulatory frameworks. In addition, environmental persistence and trace-level cross-contamination may also contribute to the occurrence of these residues and should be considered when interpreting monitoring data.

The observed distribution patterns indicate that pear samples were subjected to more intensive pesticide applications compared to apple and quince, as evidenced by higher detection frequencies, greater residue diversity, and higher MRL exceedance rates. This pattern is likely associated with crop-specific pest pressures, differences in pest management strategies, and the higher susceptibility of pear orchards to both pre- and post-harvest infestations. Variations in agronomic practices and regional differences in pesticide use patterns may have further contributed to the observed residue profiles. Furthermore, the higher residue frequencies observed in apple and pear samples may also be associated with the larger cultivation areas, greater production volumes, and more intensive commercial production systems typically employed for these commodities. These systems are frequently characterised by monoculture practices, which may increase pest and disease pressure and thus necessitate more frequent pesticide applications. The comparatively lower residue occurrence observed in quince may be attributable to the less intensive production systems generally associated with this crop.

### 2.2. Residue Profiles and Dominant Pesticides

Distinct pesticide occurrence patterns were observed across the different fruit matrices, reflecting crop-specific pest management strategies and varying agronomic practices. Acetamiprid was the most frequently detected pesticide in apples (32.7%) and was also commonly detected in pears (40%), but was not detected in quince samples. Concentration ranges were comparable between apples (0.014–0.123 mg kg^−1^) and pears (0.011–0.134 mg kg^−1^), suggesting similar application patterns across these two commodities. As a neonicotinoid insecticide, acetamiprid is widely used to control a broad spectrum of pests, including Hemiptera and Lepidoptera species [32].

Spirodiclofen, an acaricide belonging to the ketoenol class, exhibited the highest detection frequency in pears (52%), with concentrations ranging from 0.011 to 0.354 mg kg^−1^, and was also frequently detected in apples (29.1%; 0.012–0.213 mg kg^−1^), whereas it was not detected in quince samples. This compound is primarily used for the control of mite infestations and has been associated with relatively persistent residues in fruit matrices [33].

In quince samples, lambda-cyhalothrin, a pyrethroid insecticide, was identified as the dominant pesticide (20%), with concentrations ranging from 0.011 to 0.154 mg kg^−1^ (mean = 0.058 mg kg^−1^). At the same time, it was not detected in apple and pear samples. This matrix-specific occurrence suggests targeted application practices, potentially related to pest profiles unique to quince cultivation.

Difenoconazole, a systemic triazole fungicide, was among the most frequently detected pesticides in pears (48%), reflecting its extensive use in the management of fungal pathogens during both pre- and post-harvest stages in pome fruit production. Similarly, pyrimethanil, a fungicide commonly used against post-harvest pathogens, and boscalid, a carboxamide fungicide detected across all fruit types, further support the predominance of fungicide applications aimed at controlling fungal infections and extending shelf life. In quince samples, chlorantraniliprole, an anthranilic diamide insecticide, was identified as the second most frequently detected pesticide (12%), indicating its role in controlling Lepidopteran pests specific to this commodity.

Additional pesticides were detected at lower frequencies across all fruit types, including pyridaben, chlorantraniliprole, and tebufenpyrad in apples, and tebuconazole in pears. Although these compounds were less frequently detected, their presence contributes to the overall residue profile. It highlights the diversity of pesticide inputs used to manage insect pests, mites, and fungal diseases in pome fruit production systems.

### 2.3. Comparison with Previous Studies

The data presented in Table 2 provide a comparison of pesticide residue occurrence in apple, pear, and quince samples reported in Türkiye and other regions worldwide. The findings demonstrate substantial variability in both detection frequencies and residue levels, reflecting differences in agricultural practices, pesticide usage intensity, regulatory enforcement, and environmental conditions across regions.

For apple samples, pesticide detection frequencies reported in the literature vary widely, often exceeding 80% in countries such as China, the Czech Republic, Egypt, Morocco, and Poland, with some studies reporting values above 90% [17,19,20,21,22,28]. In contrast, studies conducted in Türkiye show a broader, more heterogeneous range (55–100%), indicating variability in pesticide application practices and monitoring intensity [15,18,23,24]. In the present study, a detection frequency of 76.4% and an MRL exceedance rate of 14.5% were observed, indicating that the results fall within the range reported for Türkiye. However, the exceedance rate appears higher than that reported in several previous studies. The maximum boscalid residue concentration identified in apple samples (0.295 mg kg^−1^) represents a low-to-moderate contamination level compared with the highest values reported in the literature, which can exceed 2 mg kg^−1^ in certain cases [26]. The predominance of acetamiprid is consistent with previous reports, where neonicotinoid insecticides are frequently detected in apple matrices. In contrast, the relatively high occurrence of spirodiclofen observed in the present study may reflect region-specific pest management practices.

In pear samples, the literature consistently reports high pesticide detection frequencies, generally 60–100%, while MRL exceedance rates exhibit considerable variability [25,28,30,31]. Studies conducted in Türkiye typically report detection frequencies of 60–90%, with MRL exceedance rates of 5–40% [15,23,24]. In this study, a detection frequency of 86% and an MRL exceedance rate of 30% were observed, positioning the findings at the upper range of previously reported values. Notably, the maximum residue concentration detected in pear samples (0.690 mg kg^−1^) for spinetoram indicates a moderate contamination level; however, the relatively high exceedance rate suggests more intensive or less controlled pesticide use. The diversity of frequently detected pesticides in pears, including spirodiclofen, difenoconazole, pyrimethanil, and acetamiprid, further supports the observation that pear production systems may employ more complex and intensive pest management strategies than those of other pome fruits.

In contrast, quince samples are characterised by comparatively lower pesticide contamination. Although the number of available studies is limited, existing data consistently indicate lower detection frequencies and minimal MRL exceedances. The present findings, showing a detection frequency of 30% and an MRL exceedance rate of 2%, are in close agreement with previous data reported in Türkiye [29]. Similarly, studies conducted in other regions have reported either low detection rates or no MRL exceedances, even when residues were detected in all samples [31]. The maximum residue concentration observed in this study (0.154 mg kg^−1^) for lambda-cyhalothrin further supports the relatively low residue levels in quince compared to apple and pear.

The results indicate that pesticide residues are widely detected in pome fruits across different regions; however, the magnitude of contamination and the frequency of MRL exceedances vary considerably. These differences are likely influenced by crop-specific pest pressures, pesticide application regimes, climatic conditions, and the effectiveness of regulatory control and monitoring systems. The comparatively higher exceedance rates observed in some cases highlight the need for greater adherence to good agricultural practices, particularly regarding pre-harvest intervals and the rational use of plant protection products.

### 2.4. Quality Assessment Based on the Index of Quality for Residues (IqR)

The quality and safety of agricultural commodities with respect to pesticide residues are traditionally evaluated based on compliance with established MRLs. However, the simultaneous presence of multiple residues may influence overall product quality due to potential additive or synergistic effects. The *IqR* has therefore been proposed as a complementary metric capable of identifying “low-quality” samples even when individual residue levels remain below regulatory limits [36]. This approach provides a more integrative assessment of residue levels and enables the evaluation of cumulative contamination patterns.

**Figure 2:**
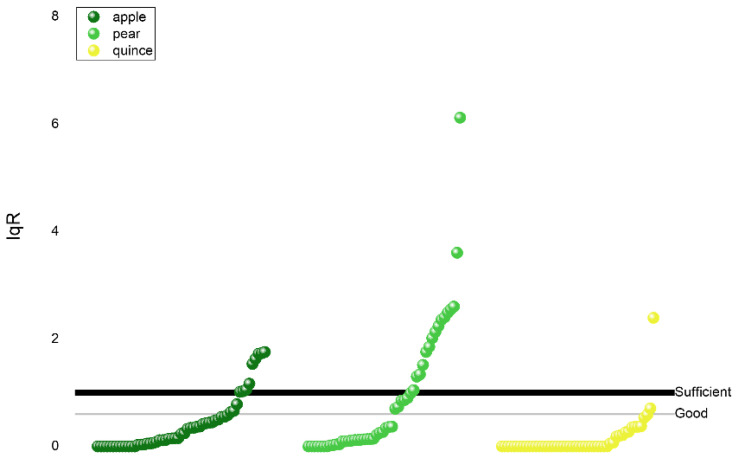
Distribution of Index of Quality for Residues (*IqR*) values in apple, pear, and quince samples, showing variability among commodities and the threshold for acceptable quality.

In the present study, the overall quality of apple, pear, and quince samples was generally satisfactory; however, notable differences were observed among the fruit types (Figure 2). Apple samples were predominantly classified as good (54.5%) or excellent (23.6%), with a smaller proportion categorised as adequate (5.5%) or inadequate (16.4%). In contrast, pear samples exhibited a less favourable quality profile, with only 14% classified as excellent and a substantially higher proportion (32%) categorised as inadequate. Intermediate categories included 42% good and 12% adequate samples, indicating a broader distribution of quality levels compared to apples. Quince samples demonstrated the most favourable quality profile, with 70% classified as excellent and 26% as good, while only 2% were categorised as adequate and 2% as inadequate. These findings indicate that, compared with apple and pear, quince samples exhibit a markedly lower cumulative residue impact.

A more detailed evaluation of *IqR* values within the inadequate category further highlights differences between fruit types. In apple samples, *IqR* values for inadequate samples ranged between 1 and 2, suggesting moderate cumulative residue contributions. In pears, however, a wider and more pronounced distribution was observed, with six samples exhibiting *IqR* values between 1 and 2, nine samples between 2 and 5, and one sample exceeding 5, reaching a maximum value of 6.1. In contrast, only a single quince sample was classified as inadequate, with an *IqR* value of 2.39.

These results clearly indicate that pear samples exhibit a higher frequency of inadequate quality classifications and higher cumulative residue levels than apple and quince samples. This pattern is consistent with the higher detection frequencies, residue diversity, and MRL exceedance rates observed in pears, suggesting that more intensive or complex pesticide application practices may contribute to the observed quality differences.

### 2.5. Risk Ranking of Pesticide Residues

**Figure 3:**
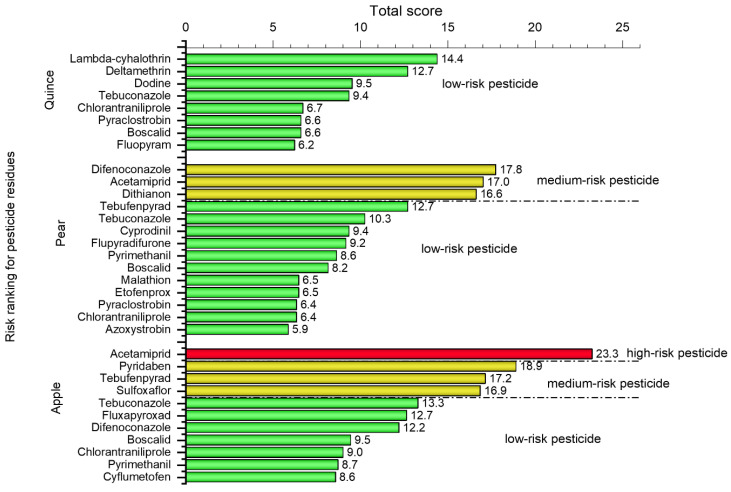
Risk ranking of detected pesticide residues in pome fruits based on cumulative scoring, illustrating their classification into low-, medium-, and high-risk categories.

The risk ranking of pesticide residues detected in apple, pear, and quince samples is shown in Figure 3. A total of 12 residues in apples, 14 in pears, and 8 in quinces were included in the ranking assessment. For pesticides not authorised for use in the EU, the application frequency parameter was excluded from the calculation, as these compounds should not be applied to these commodities under the relevant regulatory framework.

In apple samples, acetamiprid was the only pesticide classified within the high-risk category, with a total score of 23.3. This finding is notable because acetamiprid was also the most frequently detected residue in apples, indicating that both occurrence frequency and toxicological relevance contributed to its prioritisation. Three additional pesticides, pyridaben (18.9), tebufenpyrad (17.2), and sulfoxaflor (16.9), were assigned to the medium-risk category. The remaining eight pesticides were classified as low risk, with total scores below 15. These results suggest that the overall residue risk profile in apples was largely driven by a limited number of insecticides and acaricides rather than by the full spectrum of detected residues.

In pear samples, no pesticide reached the high-risk category. However, three compounds were classified as medium risk, namely difenoconazole (17.8), acetamiprid (17.0), and dithianon (16.6). The remaining 11 pesticides were assigned to the low-risk category. This pattern indicates that, although pears showed higher residue frequency, greater residue diversity, and higher MRL exceedance rates than apples and quinces, the risk ranking was distributed mainly within the low- and medium-risk categories. Therefore, the risk profile in pears appears to be associated with cumulative residue diversity and regulatory exceedance rather than the dominance of a single high-risk compound.

In quince samples, all eight evaluated pesticides were classified as low risk, with total scores below 15. This result is consistent with the lower detection frequency, limited residue diversity, and low MRL exceedance rate observed for quince samples.

Although the risk-ranking approach provides a useful framework for prioritising pesticide residues according to their potential concern, several limitations should be acknowledged. The ranking system is based on predefined scoring criteria and weighting factors, including an assigned score for potentially vulnerable population groups. It therefore represents a semi-quantitative assessment rather than a direct measure of health risk. Furthermore, the model does not explicitly account for cumulative toxicity, interactions among pesticide residues, or variability in individual consumption patterns. Therefore, the ranking results should be interpreted as a prioritisation tool intended to support monitoring and risk-management decisions rather than as a substitute for comprehensive dietary exposure and toxicological risk assessments.

### 2.6. Deterministic Dietary Exposure and Risk Characterisation

Deterministic dietary exposure was assessed for adults and children to characterise chronic and acute health risks associated with pesticide residues in apple, pear, quince, and their combined consumption as pome fruits (Table S1). When expressed using the middle bound (MB) scenario, chronic exposure levels followed a clear commodity-specific pattern, being highest for apples, followed by pears, and lowest for quinces in both population groups. In adults, estimated exposure levels ranged from 6.4 × 10^−6^ to 2.6 × 10^−5^ mg kg^−1^ bw day^−1^ for apples, from 1.2 × 10^−6^ to 1.5 × 10^−5^ mg kg^−1^ bw day^−1^ for pears, and from 3.9 × 10^−7^ to 1.2 × 10^−6^ mg kg^−1^ bw day^−1^ for quinces. A comparable pattern was observed in children, with exposure levels ranging from 7.8 × 10^−6^ to 3.1 × 10^−5^ mg kg^−1^ bw day^−1^ for apples, from 1.4 × 10^−6^ to 1.8 × 10^−5^ mg kg^−1^ bw day^−1^ for pears, and from 4.7 × 10^−7^ to 1.4 × 10^−6^ mg kg^−1^ bw day^−1^ for quinces.

Across all commodities, chronic exposure levels were consistently higher in children than in adults, reflecting their lower body weight and relatively higher food intake per unit body mass. Despite these differences, exposure levels remained within the same order of magnitude across population groups. These findings indicate that commodity type, rather than demographic group, is the primary determinant of variation in chronic dietary exposure.

Under the MB scenario, cumulative exposure levels in adults were estimated at 1.9 × 10^−4^ mg kg^−1^ bw day^−1^ for apples, 7.0 × 10^−5^ mg kg^−1^ bw day^−1^ for pears, and 6.5 × 10^−6^ mg kg^−1^ bw day^−1^ for quinces. In children, the cumulative exposure values were higher, reaching 2.3 × 10^−4^ mg kg^−1^ bw day^−1^ for apples, 8.6 × 10^−5^ mg kg^−1^ bw day^−1^ for pears, and 7.9 × 10^−6^ mg kg^−1^ bw day^−1^ for quinces. When combined consumption of pome fruits was considered, cumulative exposure increased to 2.7 × 10^−4^ mg kg^−1^ bw day^−1^ in adults and 3.2 × 10^−4^ mg kg^−1^ bw day^−1^ in children.

**Figure 4:**
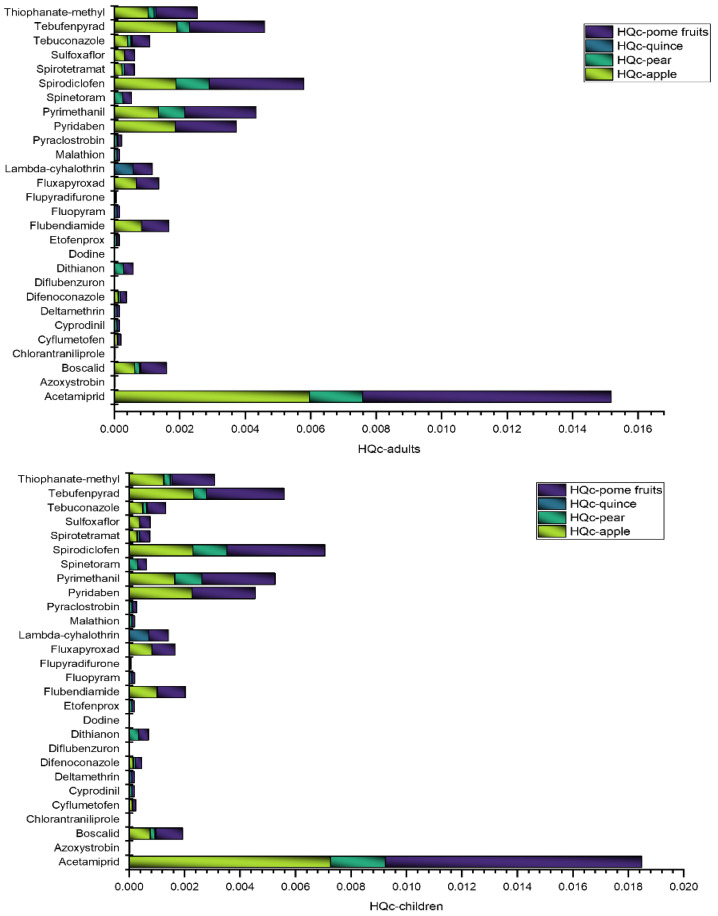
Chronic hazard quotient (*HQc*) values of individual pesticide residues in apple, pear, and quince samples, including the combined consumption scenario for pome fruits, for adult and child populations.

The calculated chronic hazard quotient (*HQc)* values (Figure 4) confirmed that chronic risks remained well below the threshold of concern. In adults, *HQc* values for individual pesticides ranged from 2.7 × 10^−6^ to 0.0044 (lower bound, LB) and from 9.3 × 10^−6^ to 0.0060 (upper bound, UB) for apples, from 4.5 × 10^−7^ to 0.0014 (LB) and from 2.3 × 10^−6^ to 0.0016 (UB) for pears, and from 2.1 × 10^−7^ to 3.5 × 10^−4^ (LB) and from 6.3 × 10^−7^ to 0.0006 (UB) for quinces. In children, *HQc* values ranged from 3.2 × 10^−6^ to 0.0053 (LB) and from 1.1 × 10^−5^ to 0.0073 (UB) for apples, from 5.4 × 10^−7^ to 0.0017 (LB) and from 2.8 × 10^−6^ to 0.0020 (UB) for pears, and from 2.5 × 10^−7^ to 0.0004 (LB) and from 7.7 × 10^−7^ to 0.0007 (UB) for quinces. These values were far below 1, indicating that chronic exposure to individual pesticide residues through the consumption of these fruits is unlikely to pose a significant health risk.

Under the worst-case scenario, chronic hazard index (*HIc*) values were estimated at 0.0174 for adults and 0.0211 for children in apple samples, 0.0053 and 0.0065 in pears, and 0.0009 and 0.0011 in quinces, respectively. These results demonstrate that cumulative chronic exposure remained substantially below the safety threshold (*HIc* < 1) for all fruit types. Acetamiprid was identified as the main contributor to *HIc* in both apples and pears, accounting for 34% and 31% of the cumulative risk, respectively, whereas lambda-cyhalothrin was the dominant contributor in quinces (65%). When combined pome fruit consumption was considered, individual *HQc* values ranged from 3.6 × 10^−5^ to 0.0167 in adults and from 4.4 × 10^−5^ to 0.0203 in children. The resulting *HIc* values reached 0.0684 for adults and 0.0833 for children, indicating that cumulative exposure through pome fruit consumption remained within acceptable limits. In this combined scenario, acetamiprid and spirodiclofen were the main contributors to *HIc*, accounting for 33% and 13%, respectively.

**Figure 5:**
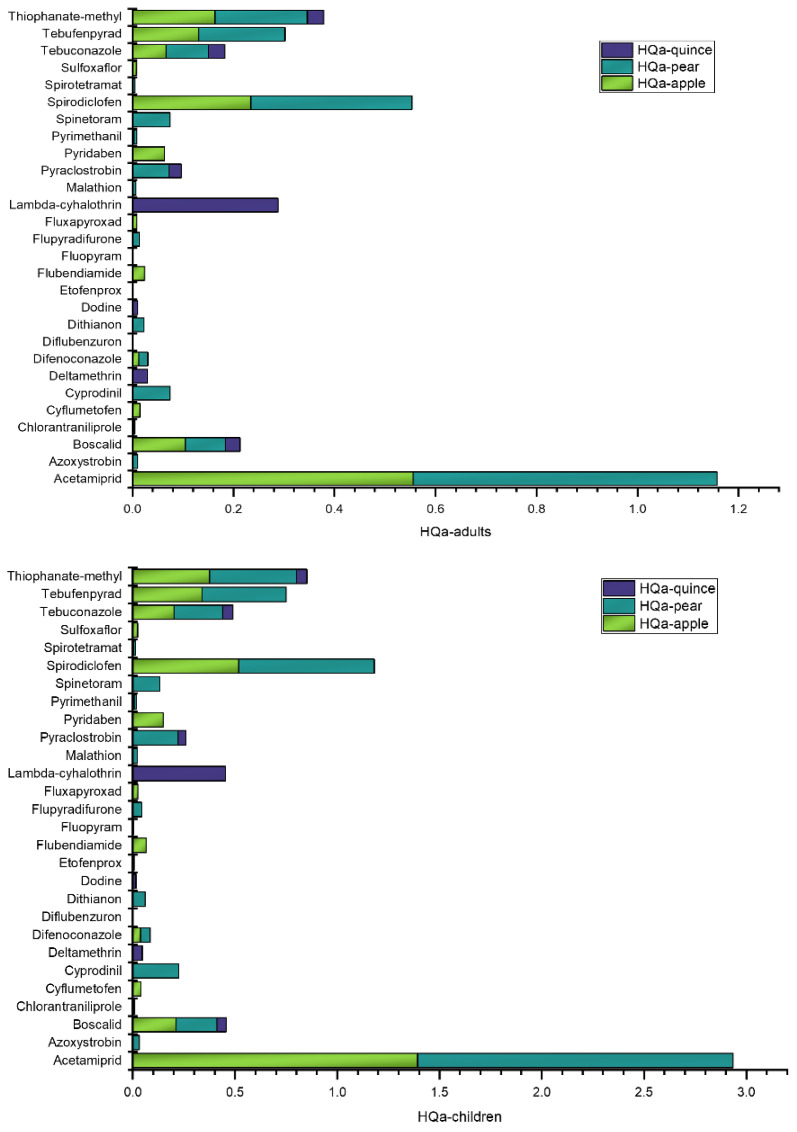
Acute hazard quotient (*HQa*) values of individual pesticide residues in pome fruits for adults and children, illustrating inter-commodity differences and highlighting compounds exceeding the safety threshold (*HQa* > 1).

In contrast to chronic exposure, a different pattern was observed for acute risk. Acute hazard quotient (*HQa*) values calculated from the highest detected residue concentrations are presented in Figure 5. In adults and children, *HQa* values ranged from 0.0015 to 0.556 and from 0.0042 to 1.39 for apples, from 0.0002 to 0.602 and from 0.0058 to 1.54 for pears, and from 0.0004 to 0.289 and from 0.0007 to 0.455 for quinces, respectively. In adults, all *HQa* values remained below 1, indicating that short-term consumption is unlikely to pose an acute health risk. However, in children, *HQa* values exceeded the threshold of 1 for acetamiprid in both apple (18 samples) and pear (20 samples) samples, highlighting a potential acute health concern under high-exposure conditions.

Comparison with previous studies further supports the distinction between chronic and acute risk patterns. Chronic risk estimates reported in the literature generally remain within safe limits, which is consistent with the present findings. For apple samples, previously reported *HIc* values ranged from 0.0002 to 0.34 in adults, while the highest value reported for children was 0.144 [17,18,26,37].

In contrast, acute risk estimates exhibit greater variability across studies and appear more sensitive to region-specific residue profiles. In the present study, acute hazard index (*HIa*) values associated with apple consumption were 1.40 for adults and 3.41 for children, exceeding the safety threshold. Similar acute risk concerns have been reported in Kazakhstan (*HIa* = 2.50 for adults and 11.7 for children) [34] and Poland (*HIa* = 22.9 for adults and 83.4 for children) [35], where substantially higher *HIa* values were observed for apple consumption. However, studies from Egypt (*HIa* = 0.578 for children) [37] and China (*HIa* = 0.542 for children) [38] reported values below 1, suggesting that acute risk levels may vary considerably depending on agricultural practices, pesticide application patterns, and residue concentrations.

A comparable trend was observed for pears, with *HIa* values of 1.75 in adults and 4.31 in children. Although some studies have reported lower values [25,26], previous findings from Türkiye also indicate that acute exposure associated with pear consumption may exceed the threshold of concern under certain conditions [23]. In contrast, acute risk estimates for quince remained below the critical threshold, with *HIa* values of 0.450 in adults and 0.709 in children, confirming its comparatively favourable residue profile.

The deterministic assessment indicates that chronic dietary exposure to pesticide residues through pome fruit consumption remains within acceptable safety limits for both adults and children. In contrast, acute exposure is a more critical endpoint, particularly for children who consume apples and pears. The approximately 2.4-fold higher acute risk observed in children highlights their increased vulnerability. These findings underscore the need for targeted risk management strategies, including stricter control of high-contributing active substances, improved adherence to pre-harvest intervals, and enhanced monitoring to protect sensitive population groups.

It should be noted that the *HI* approach applied in the present study assumes dose additivity among co-occurring pesticide residues, regardless of their specific modes of action or toxicological targets. Therefore, the calculated *HI* values provide a screening-level estimate of cumulative exposure rather than a mechanistic evaluation of combined toxicity. Potential synergistic, antagonistic, or other interactions among pesticide residues were not considered, potentially leading to cumulative toxicological responses that differ from those predicted by the current assessment framework. For this reason, the *HI* values should be interpreted as conservative indicators intended to support residue prioritisation and risk-management decisions rather than as definitive measures of cumulative health risk.

### 2.7. Probabilistic Dietary Exposure and Risk Characterisation

Probabilistic dietary exposure assessment was conducted to characterise the distribution of *HIc* associated with the consumption of apple, pear, and quince, as well as their combined intake as pome fruits, in adult and child populations. The resulting distributions exhibited a consistently right-skewed pattern across all scenarios, indicating that exposure was predominantly concentrated at lower levels, with a limited proportion of higher values extending into the upper tail of the distribution.

For apple consumption, adult *HIc* values remained predominantly within the lower exposure range, although greater variability became apparent at the upper percentiles. The 5th and 95th percentiles were estimated at 0.0008 and 0.0764, respectively (mean = 0.0273; median = 0.0056). A similar distribution pattern was observed in children, with a more pronounced exposure profile and 5th and 95th percentile values of 0.0005 and 0.0882, respectively (mean = 0.0312; median = 0.0044).

In pear samples, *HIc* values were generally lower than those observed for apples. In adults, the 5th and 95th percentiles were 0.0004 and 0.0221, respectively (mean = 0.0069; median = 0.0023), whereas corresponding values in children were 0.0003 and 0.0286 (mean = 0.0098; median = 0.0019). For quinces, *HIc* values remained consistently low across both population groups. In adults, the 5th and 95th percentiles were 3.7 × 10^−5^ and 0.0018 (mean = 0.0007; median = 0.0001), whereas in children they were 5.2 × 10^−6^ and 0.0023 (mean = 0.0011; median = 0.0001), indicating limited variability and a negligible contribution to cumulative risk.

**Figure 6:**
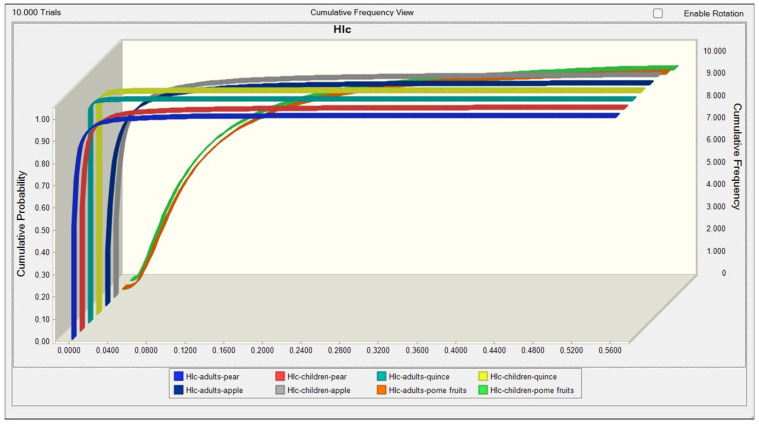
Cumulative probability distributions of probabilistic chronic risk (*HIc*) derived from Monte Carlo simulation for apple, pear, and quince samples, including the combined consumption scenario for pome fruits, for adult and child populations.

When cumulative exposure from pome fruit consumption was considered, a substantial increase in *HIc* values was observed. In adults, the 5th and 95th percentiles were estimated at 0.0189 and 0.436, respectively (mean = 0.149; median = 0.0694), whereas in children they were 0.0128 and 0.565 (mean = 0.193; median = 0.0615). The cumulative frequency curves presented in Figure 6 further support these findings. The distributions for children were consistently shifted towards higher exposure levels and exhibited a wider dispersion, indicating greater exposure across both the average and upper-bound scenarios. These results emphasise the importance of cumulative risk assessment, as exposure levels that appear limited under single-commodity evaluations become more pronounced when cumulative consumption is considered.

These findings are in line with previous probabilistic risk assessments reported in the literature and provide further insight into variability across population groups. For instance, Maleki et al. [16] reported substantially higher *HIc* values in children (0.131–0.384) compared to adults (0.015–0.077) for apple consumption, supporting the observed age-dependent differences in probabilistic risk profiles. Similarly, Zhou et al. [17] demonstrated that probabilistic risk tends to increase with decreasing age, reflecting differences in body weight and consumption patterns across population groups. In addition, previous studies have consistently shown that, despite variability in exposure distributions, chronic risk indicators generally remain below the critical threshold of 1. This pattern was also observed by Amar et al. [22], who found that *HIc* values derived from Monte Carlo simulations remained below the level of concern, even under higher-residue scenarios. These findings support the robustness of probabilistic approaches in capturing exposure heterogeneity and highlight their importance for a more realistic and comprehensive assessment of dietary risk.

## 3. Materials and Methods

### 3.1. Reagents, Chemicals, and Standards

Liquid chromatography (LC)-grade methanol and acetonitrile were obtained from Sigma-Aldrich (Steinheim, Germany). Analytical-grade glacial acetic acid (CH_3_COOH, ≥99%), ammonium formate, and formic acid were purchased from Merck KGaA (Darmstadt, Germany). QuEChERS (quick, easy, cheap, effective, rugged, and safe) extraction kits were supplied by CHROMAtific UG (Heidenrod, Germany) and consisted of extraction salts (6 g anhydrous magnesium sulphate (MgSO_4_) and 1.5 g sodium acetate (C_2_H_3_NaO_2_)) and dispersive solid-phase extraction (d-SPE) clean-up materials containing 0.4 g primary secondary amine (PSA) and 1.2 g MgSO_4_. Ultrapure water was produced using a Milli-Q purification system (Millipore, Molsheim, France).

Analytical standards of 222 pesticides (purity > 95%) were obtained from CPAchem (Sofia, Bulgaria), Sigma-Aldrich (Steinheim, Germany), and Dr. Ehrenstorfer GmbH (Augsburg, Germany). Individual primary stock solutions were prepared at a concentration of 1000 mg L^−1^ using appropriate solvents selected according to the physicochemical properties of each analyte, including polarity, ionic characteristics, and solubility (e.g., water, methanol, acetonitrile, or acetone). An intermediate mixed standard solution containing all analytes was prepared in acetonitrile at a concentration of 10 μg mL^−1^ and was subsequently used for spiking experiments and the preparation of calibration standards.

### 3.2. Sample Collection and Preparation

A total of 155 samples, including 55 apples, 50 pears, and 50 quinces, were collected from a range of retail outlets, including supermarkets (*n* = 63), local grocery stores (*n* = 51), and traditional open-air markets (*n* = 41), across different regions of Türkiye between October 2025 and March 2026. Sampling was conducted in Adana (*n* = 30), Antalya (*n* = 30), Çorum (*n* = 35), Konya (*n* = 30), and Samsun (*n* = 30), providing geographical coverage across different regions of Türkiye. Each sample (approximately 1 kg) was placed in polyethylene bags and transported to the laboratory within 24 h. Upon arrival, samples were stored under refrigerated conditions (4 °C) and analysed within three days of collection.

Before homogenisation, samples were prepared as whole commodities after removal of stems, without any prior washing or pre-treatment. In quince samples, the natural surface pubescence, when present, was gently removed manually before homogenisation. Fruits were cut into small pieces without peeling and homogenised using a laboratory-scale food grinder (Retsch GM 300, Haan, Germany). The homogenised samples were immediately subjected to extraction for pesticide residue analysis.

**Figure 7:**
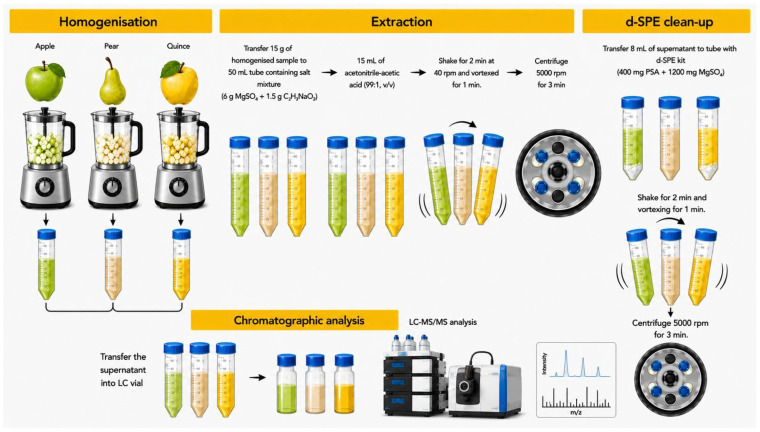
Schematic workflow of sample preparation and QuEChERS-based extraction and clean-up procedure for pesticide residue analysis in pome fruits.

Pesticide extraction was carried out using a QuEChERS-based method in accordance with AOAC Official Method 2007.01 [39], with minor modifications. The procedure involved acetonitrile extraction followed by d-SPE clean-up. The sample preparation workflow is illustrated in Figure 7.

### 3.3. LC-MS/MS Analysis and Method Validation

The determination of 222 target pesticides in pome fruit extracts was carried out using a Thermo Scientific UltiMate 3000 UHPLC system (Thermo Fisher Scientific, Bremen, Germany) coupled with a Q-Exactive Focus Orbitrap mass spectrometer (Thermo Fisher Scientific, Bremen, Germany) equipped with an electrospray ionisation (ESI) source. Chromatographic separation was achieved using an Accucore Vanquish C18 column (2.1 × 100 mm, 1.5 μm; Thermo Fisher Scientific). The mobile phase consisted of (A) water–methanol (98:2, *v*/*v*) containing 0.1% formic acid and 5 mM ammonium formate and (B) methanol–water (98:2, *v*/*v*) containing 0.1% formic acid and 5 mM ammonium formate. The gradient program was initiated at 100% A and maintained for 0.5 min, followed by a linear decrease to 30% A, then an increase to 70% B at 7.0 min. Subsequently, the composition was adjusted to 100% B at 9.0 min and maintained until 12.0 min. The system was then returned to the initial mobile phase composition at 12.1 min and re-equilibrated until 18.0 min. The flow rate was maintained at 0.3 mL min^−1^ throughout the analysis. The column temperature was set to 40 °C, and the injection volume to 10 μL. The spray voltage was 2.8 kV, while the capillary and vaporiser temperatures were set at 320 °C and 295 °C, respectively. Data acquisition and processing were performed using Xcalibur software (version 4.0, Thermo Fisher Scientific). Pesticide analysis was performed in multiple reaction monitoring (MRM) mode using two ion transitions for each analyte, and compound confirmation was achieved according to SANTE/11312/2021 v2 guidelines [40] based on retention time and ion ratio criteria. Detailed information regarding MRM transitions and collision energies is provided in Table S2.

Method validation was conducted in accordance with SANTE/11312/2021 v2 performance criteria. As apples, pears, and quinces are classified within the high-water-content commodity group according to SANTE guidance, pears were selected as the representative matrix for method validation. The validation data for all target analytes are presented in Table S3. Matrix-matched calibration was applied to compensate for matrix effects. Calibration curves were prepared at five concentration levels (0.005–0.25 mg kg^−1^) by spiking blank matrix extracts. All analytes exhibited satisfactory linearity (*R*^2^ > 0.99). Limits of quantification (LOQs) were established at 0.01 mg kg^−1^ for all compounds, ensuring adequate sensitivity for compliance with EU MRLs. Method accuracy was evaluated through recovery experiments at two spiking levels (0.01 and 0.05 mg kg^−1^), each analysed in five replicates (*n* = 5). Mean recoveries ranged from 80% to 112% for detected compounds. Precision under repeatability conditions (RSD_r_) ranged from 1.6% to 13.5%, while within-laboratory reproducibility (RSD_wR_), evaluated using ten independent determinations (*n* = 10), ranged from 3.7% to 18.6%. Expanded measurement uncertainty values (*k* = 2) for the detected pesticides ranged from 13.0% to 33.1%. All performance parameters complied with SANTE acceptance criteria (recoveries: 70–120%; precision ≤ 20%).

### 3.4. Index of Quality for Residues

The *IqR* was applied to evaluate the overall impact of multiple pesticide residues on sample quality. For each sample, the *IqR* value was calculated as the sum of the ratios between the concentration of each pesticide residue (*PRC*) and the relevant MRL, as shown in Equation (1):

(1) $$IqR=\sum_{i=0}^{n}PRCi/MRLi$$

where *i* represents each pesticide detected in the sample.

Based on the calculated *IqR* values, samples were classified into four quality categories: excellent (*IqR* = 0), good (0–0.6), adequate (0.6–1.0), and inadequate (>1.0), according to Mac Loughlin et al. [36].

### 3.5. Risk Ranking

A matrix-based risk ranking approach was applied to prioritise pesticide residues detected in pome fruit samples according to their potential toxicological relevance and exposure significance. The methodology was adapted from the framework proposed by the UK Veterinary Residues Committee [41], which combines toxicity- and exposure-related parameters into a composite risk score.

The overall risk score (S) was calculated using six individual indices representing acute toxicity (A), toxicological potency (B), dietary contribution (C), application frequency (D), vulnerability of sensitive population groups (E), and residue occurrence relative to established MRLs (F). Scoring criteria for each parameter were assigned according to the original ranking matrix (Table S4), while the calculated scores for indices A–F are presented in Table S5. The overall risk score was calculated using Equation (2)

(2) $$S=\left(A+B\right)\times \left(C+D+E\right)\times F$$

where A represents acute toxicity derived from median lethal dose (LD_50_) values [42], B corresponds to toxicological potency based on ADI values [7], C reflects dietary contribution according to pome fruit consumption data [43], D describes pesticide application frequency relative to fruit development duration, and E was assigned a value of 3 owing to limited evidence regarding highly exposed population groups. In accordance with the original UK Veterinary Residues Committee ranking framework, the highest score was assigned as a precautionary measure to account for the potential susceptibility of vulnerable consumer groups in the absence of sufficient exposure-specific information. F represents residue levels relative to the corresponding MRLs.

The frequency of pesticide application was expressed as frequency of dosing (FOD), calculated as the ratio between the number of pesticide applications (N) and the fruit growth period (P, days), as shown in Equation (3):

(3) $$FOD=\;\frac{N}{P}\;\times \;100$$

The residue-related score (F) was determined according to the distribution of measured residue concentrations relative to the corresponding MRLs using Equation (4):

(4) $$F=\frac{\left(F_{0}\;\times \;1)\;+\;{(F}_{1}\times \;2)\;+\;{(F}_{2}\;\times \;3)+\;{(F}_{3}\;\times \;4\right)}{n}$$

where F_0_ represents the number of samples without detectable pesticide residues, F_1_ corresponds to samples containing residues below the MRL, F_2_ denotes samples with residue concentrations between ≥1 and 10 times the MRL, F_3_ represents samples exceeding ≥10 times the MRL, and n is the total number of analysed samples.

### 3.6. Deterministic Risk Assessment

Deterministic dietary exposure and associated health risks were assessed for adults and children (3–10 years). The national estimated daily intake (NEDI, mg kg^−1^ bw day^−1^) was calculated using Equation (5):

(5) $$NEDI\;=\;\frac{C\;\times \;F}{bw\;}$$

where *C* represents the pesticide residue concentration (mg kg^−1^), *F* denotes the daily consumption of the relevant commodity (kg day^−1^), and *bw* is body weight (kg). Annual per capita consumption of apples, pears, and quinces in Türkiye was reported as 30.4, 5.7, and 1.9 kg, respectively, corresponding to daily consumption values of 0.083, 0.016, and 0.005 kg day^−1^ [43]. Standard body weights of 70 kg for adults and 23 kg for children were applied [44].

Left-censored data (i.e., concentrations below the LOQ) were treated using the substitution approach recommended by EFSA [45]. Lower bound (LB), upper bound (UB), and middle bound (MB) scenarios were applied by assigning values of 0, LOQ, and LOQ/2, respectively.

The chronic hazard quotient (*HQc*) for each pesticide was calculated as shown in Equation (6):

(6) $$HQc=\frac{NEDI}{ADI}$$

Acute dietary exposure was estimated using the International Estimated Short-Term Intake (IESTI) approach, following the methodology established by FAO/WHO [46]. Case 2a (Equation (7)) was applied for apples and pears, whereas Case 2b (Equation (8)) was used for quinces, reflecting differences in consumption patterns and unit weights of the commodities.

Case 2a: (25 g ≤ *Ue* < *LP*):

(7) $$IESTI=\frac{Ue\times HR\times v+\left(LP-Ue\right)\times HR}{bw}$$

Case 2b: (*Ue* > *LP*):

(8) $$IESTI=\frac{Ue\times HR\times v+\left(LP-Ue\right)\times HR}{bw}$$

where *Ue* is the unit weight of the edible portion (kg), *HR* is the highest residue level (mg kg^−1^), *v* is the variability factor, and *LP* is the large portion consumption (kg day^−1^). Commodity-specific parameters were defined as follows: apples (*Ue* = 0.26 kg; *LP* = 0.69 and 0.40 kg day^−1^ for adults and children, respectively), pears (*Ue* = 0.26 kg; *LP* = 0.68 and 0.42 kg day^−1^), and quinces (*Ue* = 0.28 kg; *LP* = 0.21 and 0.17 kg day^−1^), with a variability factor of 3 applied uniformly across all commodities [47].

Acute dietary risk was characterised using the hazard quotient (*HQa*), defined as the ratio of the IESTI to the acute reference dose (ARfD) (Equation (9)):

(9) $$HQa=\frac{IESTI}{ARfD}$$

Toxicological reference values, including the ADI and ARfD, were retrieved from the EU Pesticides Database [7]. Where ARfD values were not available, ADI values were employed as conservative surrogate thresholds for acute exposure assessment. This approach is consistent with the methodology applied by EFSA in its annual pesticide residue monitoring reports [48], in which ADI/TDI values have been used as conservative reference points when ARfD values were unavailable due to insufficient toxicological data or the absence of an acute toxicological evaluation. The resulting *HQa* values should therefore be regarded as tentative estimates of acute dietary risk and interpreted with appropriate caution.

The combined effects of multiple pesticide residues were evaluated using the hazard index (*HI*), obtained by summing individual *HQ* values (Equation (10)):

(10) HI=ΣHQ

*HQc*, *HQa*, and *HI* values greater than 1 were considered indicative of potential health concern, whereas values below this threshold were regarded as toxicologically acceptable.

### 3.7. Probabilistic Risk Assessment by Monte Carlo Simulation

Variability and uncertainty in dietary exposure were addressed through a probabilistic framework based on Monte Carlo simulation (MCS). The simulations were performed using Microsoft Excel^®^ coupled with Oracle Crystal Ball^®^ software (version 11.1.34190; Oracle, Austin, TX, USA). For each exposure scenario, 10,000 iterations were executed to obtain stable estimates of the output distributions. Residue concentrations and consumption data were described using log-normal distributions, reflecting their inherent right-skewed behaviour, whereas body weight was represented by a normal distribution. These probabilistic inputs were propagated through the exposure model to derive distributions of dietary intake and associated risk metrics.

## 4. Conclusions

Assessment of pesticide residues in pome fruits highlighted clear differences in contamination patterns among commodities, with pears exhibiting the highest levels and quinces consistently showing the lowest. The widespread occurrence of multi-residue profiles, together with the detection of non-approved compounds, indicates variability in pesticide use practices and underscores the need for strengthened regulatory control. The use of the *IqR* approach provided additional insight beyond conventional MRL-based evaluations, allowing a more integrated interpretation of sample quality. While most samples were classified within acceptable categories, the broader distribution observed in pears emphasises the importance of considering cumulative residue effects when evaluating food quality. Chronic dietary exposure was consistently found to remain within acceptable limits for both adults and children. In contrast, acute exposure proved to be a more sensitive indicator of potential concern, particularly for children, where threshold exceedances were identified for specific compounds such as acetamiprid in apples and pears. Probabilistic modelling further demonstrated that exposure variability is strongly influenced by cumulative consumption patterns and that upper-percentile risk estimates may differ substantially from deterministic outputs. The identification of acetamiprid and spirodiclofen, as dominant contributors, highlights the importance of compound-specific prioritisation within risk assessment frameworks. The results highlight the importance of cumulative risk evaluation and reinforce the need for targeted monitoring strategies focusing on high-contributing compounds, particularly to improve the protection of vulnerable population groups. It should be noted that the reported risk estimates are subject to several sources of uncertainty, including variability in food consumption patterns, residue concentrations, and the toxicological reference values used in the exposure assessment. In addition, the findings are based on samples collected during a defined monitoring period and may not fully capture temporal or regional variations in pesticide occurrence. Therefore, the deterministic and probabilistic risk estimates presented in this study should be interpreted as indicative measures of potential consumer exposure rather than definitive predictions of population-level risk.

## Figures and Tables

**Table 1:** The distribution and concentration of detected pesticides in pome fruits.

Commodity	Pesticide	EU MRL (mg kg^−1^)	No. of Detectable Samples, *n* (%)	No. of Samples > MRLs, *n* (%)	Range (mg kg^−1^)	
					Min.–Max.	Mean
Apple	Acetamiprid	0.07	18 (32.7)	7 (12.7)	0.014–0.123	0.056
	Boscalid	2	10 (18.7)	–	0.015–0.295	0.070
	Chlorantraniliprole	0.4	7 (12.7)	–	0.013–0.074	0.027
	Cyflumetofen	0.4	4 (7.3)	–	0.033–0.104	0.073
	Difenoconazole	0.8	1 (1.8)	–	0.040	0.040
	Flubendiamide *	0.9	5 (9.1)	–	0.012–0.077	0.031
	Fluxapyroxad	0.9	3 (5.5)	–	0.015–0.068	0.036
	Pyridaben	0.15	9 (16.4)	1 (1.8)	0.013–0.175	0.045
	Pyrimethanil	15	5 (9.1)	–	0.012–0.177	0.063
	Spirodiclofen *	0.8	16 (29.1)	–	0.012–0.213	0.057
	Spirotetramat *	0.7	1 (1.8)	–	0.026	0.026
	Sulfoxaflor	0.4	3 (5.5)	–	0.014–0.027	0.020
	Tebuconazole	0.3	1 (1.8)	–	0.028	0.028
	Tebufenpyrad	0.3	6 (10.9)	–	0.033–0.105	0.066
	Thiophanate-methyl *	0.5	4 (7.3)	–	0.062–0.183	0.111
Pear	Acetamiprid	0.07	20 (40)	10 (20)	0.011–0.134	0.076
	Azoxystrobin	0.01	1 (2)	1 (2)	0.020	0.020
	Boscalid	1.5	18 (36)	–	0.014–0.157	0.056
	Chlorantraniliprole	0.4	3 (6)	–	0.031–0.271	0.116
	Cyprodinil	2	2 (4)	–	0.019–0.039	0.029
	Difenoconazole	0.8	24 (48)	–	0.011–0.110	0.047
	Dithianon	1.5	2 (4)	–	0.065–0.102	0.084
	Etofenprox	0.7	1 (2)	–	0.031	0.031
	Flupyradifurone	0.6	1 (2)	–	0.029	0.029
	Malathion	0.02	4 (8)	3 (6)	0.014–0.029	0.022
	Pyraclostrobin	0.5	3 (6)	–	0.017–0.031	0.022
	Pyrimethanil	15	22 (44)	–	0.013–0.479	0.094
	Spinetoram *	0.15	3 (6)	2 (4)	0.032–0.690	0.334
	Spirodiclofen *	0.8	26 (52)	–	0.011–0.354	0.122
	Spirotetramat *	0.7	2 (4)	–	0.080–0.156	0.118
	Tebuconazole	0.3	7 (14)	–	0.014–0.073	0.037
	Tebufenpyrad	0.3	3 (6)	–	0.062–0.184	0.128
	Thiophanate-methyl *	0.5	4 (8)	–	0.016–0.215	0.101
Quince	Boscalid	1.5	5 (10)	–	0.014–0.121	0.049
	Chlorantraniliprole	0.4	6 (12)	–	0.017–0.073	0.037
	Deltamethrin	0.1	3 (6)	–	0.012–0.032	0.020
	Diflubenzuron *	0.01	1 (2)	1 (2)	0.014	0.014
	Dodine	5	3 (6)	–	0.027–0.113	0.073
	Fluopyram	0.8	2 (4)	–	0.078–0.110	0.094
	Lambda-cyhalothrin	0.2	10 (20)	–	0.011–0.154	0.058
	Pyraclostrobin	0.5	5 (10)	–	0.018–0.078	0.043
	Tebuconazole	0.5	2 (4)	–	0.043–0.105	0.074
	Thiophanate-methyl *	0.5	4 (8)	–	0.014–0.068	0.042

* Not approved in the EU.

**Table 2:** Comparison of pesticide residue studies on apples, pears, and quinces.

Fruit	Region	No. of Samples (*n*)	Pesticides (Screened/ Detected)	Samples with Residues, *n* (%)	Samples > MRL, *n* (%)	Maximum Level, mg kg^−1^ (Pesticide)	Most Frequently Detected Pesticides ^a^	Ref
Apple	China	120	17/6	110 (91.7)	9 (7.5)	0.145 (tebuconazole)	Thiamethoxam, carbendazim	[17]
Apple	China	133	102/34	128 (96.2)	0	0.523 (tebuconazole)	Carbendazim, tebuconazole, acetamiprid	[20]
Apple	Czech Republic	265	460/74	260 (98.1)	4 (1.5)	0.323 (folpet)	Captan, difenoconazole, acetamiprid	[21]
Apple	Egypt	10176	430/– ^b^	9962 (97.9)	– (8.6–28.6)	0.175 (lambda-cyhalothrin)	Acetamiprid, cypermethrin	[19]
Apple	Jordan	30	178/2	10 (33.3)	6 (20)	2.94 (pyrimethanil)	Tebuconozole	[26]
Apple	Kazakhstan	80	426/24	39 (49)	–	0.623 (propargite)	Chlorpyrifos, cypermethrin	[34]
Apple	Morocco	31	22/22	26 (83)	–	0.43 (carbendazim)	Carbendazim, indoxacarb	[22]
Apple	Poland	1223	535/57	785 (64.2)	41 (3.4)	1.00 (chlorpyrifos-methyl)	Captan, pirimicarb	[35]
Apple	Poland	89	481/36	73 (82)	1 (1.1)	2.40 (fosetyl-Al)	Captan, flonicamid, acetamiprid	[28]
Apple	Romania	42	74/17	42 (100)	0	0.257 (boscalid)	Boscalid, acetamiprid, pyraclostrobin	[31]
Apple	Serbia	351	–	225 (64.1)	12 (3.4)	–	–	[30]
Apple	Türkiye	20	393/–	11 (55)	0	–	Thiacloprid, acetamiprid	[15]
Apple	Türkiye	17	260/11	13 (76.5)	8 (47)	0.485 (diflubenzuron)	Dimethoate	[24]
Apple	Türkiye	11	38/–	11 (100)	–	0.057 (diflubenzuron)	–	[23]
Apple	Türkiye	100	225/15	64 (64)	11 (11)	0.156 (diflubenzuron)	Thiophanate-methyl, chlorantraniliprole	[18]
Pear	China	21	68/16	21 (100)	1 (4.8)	0.210 (prochloraz)	Pyraclostrobin, chlorantraniliprole	[25]
Pear	Jordan	24	178/2	2 (8.3)	2 (8.3)	0.160 (fludioxonil)	Fludioxonil, pyridaben	[26]
Pear	Poland	72	479/29	62 (86.1)	1 (1.4)	1.10 (captan)	Captan, acetamiprid	[28]
Pear	Romania	20	74/13	21 (100)	0	0.469 (pyrimethanil)	Thiacloprid	[31]
Pear	Serbia	95	–	68 (71.6)	6 (6.3)	–	–	[30]
Pear	Türkiye	39	395/–	26 (66.6)	2 (5.1)	–	–	[29]
Pear	Türkiye	10	260/5	6 (60)	4 (40)	0.269 (dimethoate)	Dimethoate, boscalid, cypermethrin	[24]
Pear	Türkiye	11	38/13	10 (90.9)	1 (9.1)	0.065 (pyriproxyfen)	–	[23]
Quince	Romania	5	74/6	5 (100)	0	0.224 (carbendazim	Acetamiprid, difenoconazole, carbendazim	[31]
Quince	Türkiye	90	395/–	24 (34)	2 (2.2)	–	–	[29]

^a^ The highest measured concentration refers to the highest value detected among all pesticides analysed in the study. ^b^ –: Not available.

## Data Availability

The original contributions presented in the study are included in the article and Supplementary Material, further inquiries ca be directed to the corresponding authors.

## Supplementary Materials

The following supporting information can be downloaded at: https://www.mdpi.com/article/10.3390/molecules31122132/s1, Table S1: Estimated dietary exposure and hazard quotients (*HQc* and *HQa*) associated with pesticides detected in apples, pears, and quinces for adult and child populations; Table S2: MS/MS parameters (precursor ions, product ions, and collision energies) for 222 target pesticides analysed by LC-MS/MS; Table S3: In-house validation data for 222 pesticides analysed in pome fruits by LC-MS/MS; Table S4: Definition and individual scores of indices for the pesticide residual risk ranking; Table S5: Assigned scores for indices A-F used in the pesticide residue risk scoring system.
