# Supplementary material for: Pesticide Residues in Pome Fruits: Occurrence, Quality Profiling, and Advanced Dietary Risk Characterisation
Source: Molecules. 2026 Jun 17;31(12):2132. doi: 10.3390/molecules31122132 (PMC13305187; doi:10.3390/molecules31122132)
Supplement: Supplementary file 1 [file molecules-31-02132-s001.zip › molecules-4359231-supplementary.pdf]

Supplementary Material

Pesticide residues in pome fruits: Occurrence, quality profiling, and advanced dietary risk characterisation

Nimo Hussein Yussuf [1,2], Tuba Buyuksirit-Bedir [2], Cagla Kayisoglu [3], Eylem Odabas [2], Fatma Oznur Afacan [4], Ozgur Golge [5], Tamara Lazarević-Pašti [6,*] and Bulent Kabak [2,*]

[1] Department of Nutrition and Food Science, Edna Adan University, Iftin Road, Hargeisa 19030, Somaliland, Somalia; qadanbudhle@gmail.com
[2] Department of Food Engineering, Faculty of Engineering and Natural Sciences, Hitit University, Corum 19030, Türkiye; tubabuyuksirit@hitit.edu.tr (T.B.-B.); eylemodabas19@gmail.com (E.O.)
[3] Scientific Technical Application and Research Center, Hitit University, Corum 19030, Türkiye; caglakayisoglu@hitit.edu.tr
[4] Department of Nutrition and Dietetics, Hamidiye Faculty of Health Sciences, University of Health Sciences, Istanbul 34668, Türkiye; ftmznr@gmail.com
[5] Department of Gastronomy and Culinary Arts, Faculty of Tourism, Alanya Alaaddin Keykubat University, Alanya 07425, Türkiye; ozgur.golge@alanya.edu.tr
[6] Department of Physical Chemistry, VINČA Institute of Nuclear Sciences—National Institute of the Republic of Serbia, University of Belgrade, 11000 Belgrade, Serbia
* Correspondence: tamara@vin.bg.ac.rs (T.L.-P.); bulentkabak@hitit.edu.tr (B.K.)

Table S1. Estimated dietary exposure and hazard quotients (*HQc* and *HQa*) associated with pesticides detected in apples, pears, and quinces for adult and child populations.

Pesticide	Apple				Pear				Quince				Pome fruits	
	Adults		Children		Adults		Children		Adults		Children		Adults	Children
	HQc (UB[a])	*HQa*	*HQc (UB)*	*HQa*	*HQc (UB)*	*HQa*	*HQc (UB)*	*HQa*	*HQc (UB)*	*HQa*	*HQc (UB)*	*HQa*	*HQc (UB)*	*HQc*
Acetamiprid	0.0060	0.556	0.0073	1.39	0.0016	0.602	0.0020	1.54					0.0167	0.0203
Azoxystrobin					0.0000	0.0104	1.4×10^{-5}	0.0328					0.0001	0.0001
Boscalid	0.0006	0.105	0.0008	0.214	0.0002	0.0799	0.0002	0.199	2.6×10^{-5}	0.0283	3.2×10^{-5}	0.0446	0.0021	0.0025
Chlorantraniliprole	1.0×10^{-5}	0.0015	1.1×10^{-5}	0.0042	2.3×10^{-6}	0.0026	2.8×10^{-6}	0.0058		0.0004	7.6×10^{-7}	0.0007	3.6×10^{-5}	4.4×10^{-5}
Cyflumetofen	0.0001	0.0154	0.0001	0.0400									0.0001	0.0001
Cyprodinil					0.0001	0.0747	0.0001	0.225					0.0005	0.0006
Deltamethrin									6.3×10^{-7}	0.0300	0.0001	0.0472	0.0014	0.0017
Difenoconazole	0.0001	0.0131	0.0002	0.0388	0.0001	0.0176	0.0001	0.0467					0.0005	0.0006
Diflubenzuron									7.9×10^{-5}	0.0013	9.1×10^{-6}	0.0021	0.0001	0.0002
Dithianon					0.0003	0.0229	0.0004	0.0616					0.0018	0.0021
Dodine									7.5×10^{-6}	0.0106	1.2×10^{-5}	0.0167	0.0002	0.0002
Etofenprox					0.0001	0.0022	0.0001	0.0067					0.0005	0.0006
Flubendiamide	0.0008	0.0240	0.0010	0.0655									0.0009	0.0012
Fluopyram									1.0×10^{-5}	0.0021	0.0001	0.0032	0.0015	0.0018
Flupyradifurone					4.0×10^{-5}	0.0144	4.4×10^{-5}	0.0444					0.0002	0.0003
Fluxapyroxad	0.0007	0.0093	0.0008	0.0259									0.0008	0.0009
Lambda-cyhalothrin									8.3×10^{-5}	0.289	0.0007	0.4545	0.0106	0.0129
Malathion					0.0001	0.0072	0.0001	0.0222					0.0005	0.0006
Pyraclostrobin					0.0001	0.0726	0.0001	0.223	3.3×10^{-5}	0.0244	4.0×10^{-5}	0.0384	0.0011	0.0013
Pyridaben	0.0019	0.0642	0.0023	0.149									0.0021	0.0026
Pyrimethanil	0.0014	0.0032	0.0017	0.0075	0.0008	0.0058	0.0010	0.0112					0.0064	0.0078
Spinetoram					0.0003	0.0750	0.0003	0.133					0.0016	0.0019
Spirodiclofen	0.0019	0.235	0.0023	0.520	0.0010	0.319	0.0012	0.663					0.0083	0.0101
Spirotetramat	0.0002	0.0020	0.0003	0.0061	0.0001	0.0032	0.0001	0.0079					0.0007	0.0008
Sulfoxaflor	0.0003	0.0080	0.0004	0.0244									0.0004	0.0004
Tebuconazole	0.0004	0.0666	0.0005	0.203	0.0001	0.0839	0.0001	0.237	3.1×10^{-5}	0.0328	3.8×10^{-5}	0.0516	0.0017	0.0020
Tebufenpyrad	0.0019	0.132	0.0023	0.340	0.0004	0.171	0.0005	0.411					0.0045	0.0055
Thiophanate-methyl	0.0010	0.164	0.0013	0.376	0.0002	0.183	0.0002	0.427	4.7×10^{-5}	0.0318	0.0001	0.0502	0.0032	0.0039

[a] UB: Upper bound (results below the LOQ were replaced with the value of LOQ)

Table S2. MS/MS parameters (precursor ions, product ions, and collision energies) for 222 target pesticides analysed by LC-MS/MS.

Pesticide	Precursor ion (*m/z*)	Product ion (*m/z*)	CE[a] (eV)
2.4-D	219.2	125.0	27
	219.2	161.0	15
2.4-Dimethylaniline	122.1	77.2	-31
	122.1	107.1	-23
2-phenylphenol	170.1	170.1	22
	155.0	141.0	34
Acephate	184.1	125.1	18
	184.1	143.0	8
Acetamiprid	223.0	56.1	15
	223.0	126.0	20
Acibenzolar-s-methyl	211.0	136.1	41
	211.0	91.1	29
Alachor	219.0	163.0	5
	219.0	161.0	5
Aldicarb	208.2	89.1	19
	208.2	116.1	8
Aldicarb sulfone	223.0	86.0	14
	223.0	148.0	10
Aldicarb sulfoxide	207.0	89.0	14
	207.0	132.0	10
Ametoctradin	276.3	149.1	39
	276.3	176.2	42
Amitraz	294.4	163.2	14
	294.4	253.3	12
Atrazine	216.1	96.1	23
	216.1	174.1	18
Azinphos ethyl	346.0	132.1	-20
	346.0	160.1	-11
Azinphos-methyl	317.9	159.9	6
	317.9	260.9	6
Azoxystrobin	404.0	329.0	30
	404.0	372.0	15
Benalaxyl	326.2	148.1	-23
	326.2	208.0	-17
Benfuracarb	411.2	194.9	-25
	411.2	252.0	-15
Bensulfuron-methyl	411.1	149.0	22
	411.1	182.0	20
Bentazone	238.9	175.0	18
	238.9	196.9	19
Bifenazate	301.0	198	11
	301.0	170	22
Bifenthrin	440.2	166.3	47
	440.2	181.3	13
Boscalid	342.9	139.9	20
	342.9	307.0	20
Bromoxynil	275.8	78.7	17
	275.8	80.6	17
Bromuconazole	376.0	70.1	25
	376.0	158.9	35

Bupirimate	317.0	108.0	28
	317.0	166.0	28
Buprofezin	306.1	57.4	20
	306.1	201.0	12
Cadusafos	271.1	131.0	22
	271.1	159.0	16
Carbaryl	202.0	117.0	28
	202.0	145.0	22
Carbendazim	192.1	132.1	28
	192.1	160.1	18
Carbofuran	222.1	123.0	16
	222.1	165.1	13
Carbosulfan	381.0	118.0	15
	381.0	160.0	10
Carboxin	236.0	87.0	22
	236.0	143.0	16
Chlorantraniliprole	484.0	452.9	-17
	482.0	284.0	-14
Chlorbromuron	295.1	206.0	-20
	293.1	182.1	-17
Chlorfluazuron	539.8	158.0	20
	539.8	382.9	20
Chloridazon	222.0	77.0	30
	222.0	92.0	30
Chlorpyrifos	350.0	197.8	25
	350.0	97.1	32
Chlorpyrifos-methyl	322.1	125.1	35
	322.1	290.0	20
Chlorsulfuron	358.0	141.0	16
	358.0	167.0	18
Clethodim	360.0	164.0	18
	360.0	268.1	12
Clodinafop	350.0	91.0	32
	350.0	266.0	16
Clofentezine	303.0	102.0	35
	303.0	138.0	22
Clothianidin	250.0	132.0	18
	250.0	169.0	12
Cyazofamid	325.2	108.1	14
	325.1	261.1	10
Cycloate	216.0	134.0	6
	216.0	154.0	4
Cyflumetofen	465.2	173.0	24
	465.2	249.0	16
Cymoxanil	199.0	111.0	18
	199.0	128.0	8
Cypermethrin (gc)	181.0	152.0	25
	181.0	127.0	35
Cyproconazole	292.2	70.2	18
	292.2	125.1	24
Cyprodinil	226.0	93.0	33
	226.0	108.0	25
Dazomet	162.9	89.8	9
	162.9	119.9	14
	505.9	93.2	46

Deltamethrin	505.9	280.9	12
Demeton-S-methyl	231.1	61.2	30
	231.1	81.9	10
Demeton-S-methyl-sulfone	263.0	121.0	17
	263.0	169.0	17
Demeton-S-methyl-sulfoxide	247.0	109.0	25
	247.0	169.0	14
Desmedipham	301.0	136.0	22
	301.0	182.0	10
Diafenthiuron	385.4	278.3	33
	385.4	329.3	19
Diazinon	305.0	97.0	35
	305.0	169.0	17
Dichlofluanid	333.0	123.0	24
	333.0	224.0	10
Dichlorvos	221.0	79.0	34
	221.0	109.0	22
Dicrotophos	238.0	112.0	10
	238.0	193.0	10
Diethofencarb	268.0	124.0	40
	268.0	226.0	10
Difenoconazole	406.0	111.1	60
	406.0	251.1	25
Diflubenzuron	309.0	155.9	11
	309.0	289.0	10
Dimethenamid	276.0	168.0	26
	276.0	244.0	14
Dimethoate	230.1	125.0	20
	230.1	199.0	10
Dimethomorph	388.1	165.0	30
	388.1	300.9	20
Dinocap	295.1	209.0	31
	295.1	193.4	28
Dinotefuran	203.1	129.1	-15
	203.1	157.2	-12
Dithianon	296.8	263.9	14
	296.8	238.0	18
Diuron	233.0	46.3	14
	233.0	72.1	18
Dodine	228.0	57.1	23
	228.0	60.1	23
Epoxiconazole	330.0	101.0	50
	330.0	121.0	22
Ethiofencarb	226.1	107.0	17
	226.1	164.0	8
Ethofumesate	287.1	121.1	15
	287.1	259.1	10
Etofenprox	394.3	106.9	43
	394.3	177.0	15
Etoxazole	360.5	141.2	28
	360.5	177.3	20
Famoxadone	392.2	238.0	20
	392.2	331.1	10
	312.1	92.0	25

Fenamidone	312.1	236.1	14
Fenarimol	331.0	81.0	34
	331.0	268.0	22
Fenazaquin	307.2	57.2	25
	307.2	161.0	19
Fenbuconazole	337.0	70.1	20
	337.0	125.0	36
Fenhexamid	302.1	55.3	38
	302.1	97.2	22
Fenoxaprop-P-ethyl	362.1	121.0	27
	362.1	287.9	18
Fenoxycarb	302.1	88.0	20
	302.1	116.1	11
Fenpropathrin	350.1	97.0	34
	350.1	125.0	14
Fenpyroximate	422.2	138.1	32
	422.2	366.1	15
Fenthion	279.1	169.1	16
	279.1	247.1	13
Flonicamid	230.0	202.9	18
	230.1	148.1	28
Fluazinam	463.0	398.0	20
	463.0	415.9	19
Flubendiamide	683.0	408	-11
	700.0	408	-21
Fludioxonil	247.0	180.0	28
	247.0	126.0	35
Flufenoxuron	489.1	141.0	46
	489.1	158.0	22
Fluopicolide	382.9	173.0	24
	382.9	144.9	55
Fluopyram	397.1	172.9	33
	397.1	207.9	23
Flupyradifurone	289.1	126.1	-23
	289.1	291.1	-23
Flutriafol	302.1	70.2	18
	302.1	123.1	29
Fluxapyroxad	382.0	362.0	10
	382.0	342.0	20
Fonofos	247.1	109.0	20
	247.1	137.0	10
Formetanate	222.0	46.0	26
	222.0	165.0	15
Fosthiazate	284.1	104	22
	284.1	227.9	12
Furathiocarb	383.2	194.9	18
	383.2	252.0	12
Haloxyfop	434.0	91.0	34
	434.0	316.0	20
Haloxyfop-P-methyl	376.0	316.1	32
	376.0	288.0	25
Heptenophos	251.0	125.0	14
	251.0	127.0	22
	314.0	70.1	28

Hexaconazole	314.0	159.0	28
Hexaflumuron	459.0	276.0	19
	459.0	439.0	12
Hexythiazox	353.0	168.1	26
	353.0	228.1	14
Imazalil	297.0	69.0	22
	297.0	159.0	22
Imidacloprid	256.1	175.1	20
	256.1	209.1	15
Indoxacarb	528.1	203.0	36
	528.1	150.0	16
Isoprocarb	210.8	95.0	-22
	210.8	137.0	-16
Isopyrazam	360.2	244.1	-27
	360.2	340.3	-14
Ioxynil	369.8	127.1	31
	369.8	215.1	30
Iprodione	330.0	245.0	10
	330.0	288.0	5
Iprovalicarb	320.8	119.1	-23
	320.8	203.1	-11
Kresoxim-methyl	314.0	116.0	25
	314.0	206.0	40
Lambda-cyhalothrin	467.0	141.0	45
	467.0	225.0	10
Lenacil	235.2	136.1	32
	235.2	153.1	16
Linuron	249.1	160.1	18
	249.1	181.1	16
Lufenuron	509.2	175.0	40
	509.2	326.1	22
Malathion	331.0	99.0	24
	331.0	127.0	12
Malaoxon	315.0	98.9	24
	315.0	127.0	12
Mandipropamid	412.0	328.0	-17
	412.0	356.1	-12
Mecarbam	330.0	97.0	35
	330.0	227.1	8
Metalaxyl	280.3	192.4	19
	280.3	220.4	14
Metamitron	203.1	104.0	22
	203.1	175.1	16
Methacrifos	241.1	125.0	20
	241.1	209.1	8
Methamidophos	142.0	93.9	13
	142.0	124.9	13
Methiocarb	226.0	169.0	22
	226.0	164.0	10
Methomyl	162.9	87.9	9
	162.9	105.9	10
Metolachlor	284.0	176.0	20
	284.0	252.0	8
	215.0	89.0	20

Metribuzin	215.0	131.0	18
Mevinphos	225.1	127.1	15
	225.1	193.1	8
Monocrotophos	224.1	98.1	12
	224.1	127.1	16
Monolinuron	215.0	99.0	34
	215.0	126.0	22
Myclobutanil	289.1	70.2	18
	289.1	125.1	32
Nicosulfuron	411.0	106.0	32
	411.0	182.0	22
Nuarimol	315.0	81.1	28
	315.0	252.0	22
Omethoate	214.1	125.1	22
	214.1	183.1	11
Oxadixyl	279.0	132.0	34
	279.0	219.0	10
Oxamyl	237.0	72.0	10
	237.0	90.0	10
Oxyfluorfen	362.0	237.0	20
	362.0	316.0	5
Paraoxon-ethyl	276.4	174.3	25
	276.4	220.3	14
Penconazole	284.0	70.1	16
	284.0	159.0	34
Pendimethalin	282.2	194.1	17
	282.2	212.2	10
Phenmedipham	301.0	136.0	22
	301.0	168.0	10
Phenthoate	321.0	135.0	20
	321.0	163.0	12
Phorate	261.0	74.9	8
	261.0	170.9	12
Phosalone	367.9	110.9	42
	367.9	181.9	14
Phosphamidon	300.1	127.1	25
	300.1	174.1	14
Phosmet	318.0	77.0	46
	318.0	160.0	22
Pirimicarb	239.1	72.0	18
	239.1	182.1	15
Pirimiphos-ethyl	334.2	198.1	19
	334.2	182.1	19
Pirimiphos-methyl	306.0	108.0	31
	306.0	164.0	19
Prochloraz	376.2	70.1	34
	376.2	308.1	11
Profenofos	373.0	97.0	35
	373.0	303.0	15
Profoxydim	46.5	180.3	26
	46.5	280.3	24
Promecarb	208.1	109.0	15
	208.1	151.0	9
	242.0	158.0	25

Prometryn	242.0	200.1	17
Propamocarb	189.1	102.0	17
	189.1	144.0	22
Propaquizafop	444.2	100.0	20
	444.2	163.1	60
Propargite	368.4	57.1	20
	368.4	231.3	8
Propazine	230.2	146.1	24
	230.2	188.1	18
Propiconazole	342.0	69.0	22
	342.0	159.0	34
Propoxur	210.0	111.0	16
	210.0	168.0	10
Propyzamide	256.1	173.0	23
	256.1	190.0	16
Prosulfuron	418.1	139.1	22
	418.1	252.1	14
Prothiofos	345.1	133.0	56
	345.1	241.0	18
Pymetrozine	218.0	79.0	30
	218.0	105.0	20
Pyraclostrobin	388.0	194.0	-14
	388.0	162.9	-28
Pyrazophos	374.0	194.0	33
	374.0	222.0	17
Pyridaben	365.1	147.1	24
	365.1	309.1	12
Pyridaphenthion	341.0	92.0	34
	341.0	189.0	22
Pyridate	379.0	207.0	18
	379.0	351.1	10
Pyrimethanil	199.8	107.1	28
	199.8	82.2	30
Pyriproxyfen	322.1	96.0	14
	322.1	227.1	14
Rimsulfuron	431.9	182.1	22
	431.9	325.1	14
Sethoxydim	328.0	178.0	22
	328.0	282.0	10
Simazine	202.0	96.0	22
	202.0	124.0	16
Spinosyn A	732.6	98.1	59
	732.6	142.0	31
Spinosyn D	746.5	98.1	53
	746.5	142.0	31
Spinetoram	748.4	142.1	-37
	748.4	98.2	-55
Spirodiclofen	411.2	71.2	22
	413.1	315.1	14
Spiromesifen	371.1	273.2	12
	371.1	255.2	26
Spirotetramat	374.1	302.1	-18
	374.1	216	-37
	302.1	216	-30

Spirotetramat-enol	302.1	116.9	-42
Spiroxamine	298.0	100.0	32
	298.0	144.0	20
Sulfoxaflor	278.0	174.0	-10
	278.0	105.1	-13
Tau-fluvalinate	503.0	181.0	30
	503.0	208.0	5
Tebuconazole	308.0	70.1	22
	308.0	125.0	40
Tebufenozide	353.1	133.0	20
	353.1	297.1	8
Tebufenpyrad	334.0	117.0	34
	334.0	145.0	28
Teflubenzuron	379.0	339	11
	379.0	358.9	6
Tepraloxydim	342.1	166.1	20
	342.1	250.1	12
Terbufos	289.0	57.2	22
	289.0	103.0	8
Terbuthylazine	230.0	96.0	28
	230.0	174.0	16
Terbuthylazine-desethyl	202.1	79.1	26
	202.1	146.1	16
Terbutryn	242.0	68.0	45
	242.0	186.0	13
Tetraconazole	372.0	70.1	20
	372.0	159.0	30
Thiabendazole	202.0	131.0	30
	202.0	175.0	25
Thiacloprid	253.0	90.1	40
	253.0	126.0	20
Thiamethoxam	292.0	132.0	22
	292.0	211.2	12
Thifensulfuron-methyl	388.0	56.0	40
	388.0	167.0	15
Thiobencarb	258.2	89.1	48
	258.2	125.1	18
Thiodicarb	355.0	87.9	16
	355.0	107.9	16
Thiophanate-methyl	343.0	93.0	46
	343.0	151.0	22
Tolyfluanid	364.0	137.0	25
	364.0	238.0	1
Tralkoxydim	330.2	138.1	19
	330.2	284.3	13
Triadimefon	294.1	69.3	20
	294.1	197.2	15
Triadimenol	296.1	70.2	10
	296.1	99.1	15
Triallate	304.0	86.0	18
	304.0	142.9	28
Triasulfuron	402.0	141.0	20
	402.0	167.1	17
	257.0	79.0	30

Trichlorfon	257.0	109.0	18
Trifloxystrobin	409.0	145.0	40
	409.0	186.0	16
Triflumizole	346.0	73.1	10
	346.0	277.9	10
Triflumuron	356.9	154.0	13
	356.8	84.7	45
Triticonazole	318.1	70.1	16
	318.1	124.9	35
Valifenalate	399.2	154.9	40
	401.2	116.0	29
Vamidothion	288.0	118.1	28
	288.0	146.0	10
Zoxamide	336.1	186.9	-23
	336.1	158.9	-46

[a]CE: Collision energy (eV)

Table S3. In-house validation data for 222 pesticides analysed in pome fruits by LC-MS/MS.

Pesticide	LOQ (mg kg^{-1})	Recovery (%)		Repeatability (%RSD$_r$, n=5)		Within-laboratory reproducibility (%RSD$_{wR}$, n=10)		*U* (%)
		0.01 mg kg^{-1}	0.05 mg kg^{-1}	0.01 mg kg^{-1}	0.05 mg kg^{-1}	0.01 mg kg^{-1}	0.05 mg kg^{-1}	
2.4-D	0.01	110.0	98.6	2.7	3.1	13.0	4.8	20.2
2.4-Dimethylaniline	0.01	89.8	96.0	7.9	3.3	15.3	3.0	23.1
2-phenylphenol	0.01	91.4	93.9	6.9	4.6	5.3	5.5	18.2
Acephate	0.01	107.3	95.4	6.7	2.1	14.8	2.7	21.8
Acetamiprid	0.01	86.1	102.4	10.4	2.3	10.9	6.9	19.1
Acibenzolar-s-methyl	0.01	86.5	110.7	14.2	5.0	8.7	3.5	15.2
Alachor	0.01	109.9	104.2	6.7	3.1	8.4	5.8	15.3
Aldicarb	0.01	114.7	101.4	2.2	5.5	19.3	3.3	28.0
Aldicarb sulfone	0.01	111.4	103.1	3.0	6.0	4.1	7.6	14.0
Aldicarb sulfoxide	0.01	98.3	98.9	8.1	3.9	8.1	5.1	14.4
Ametoctradin	0.01	108.2	92.3	2.9	1.3	14.3	6.7	22.8
Amitraz	0.01	111.7	102.5	4.9	5.9	15.9	11.5	28.0
Atrazine	0.01	98.0	102.2	10.0	3.1	7.7	14.3	23.8
Azinphos ethyl	0.01	105.5	96.4	9.1	1.4	9.7	2.9	15.6
Azinphos-methyl	0.01	105.6	109.6	9.2	0.7	10.3	5.7	17.3
Azoxystrobin	0.01	95.2	103.7	11.5	1.5	14.0	10.3	25.4
Benalaxyl	0.01	113.7	101.4	2.3	3.9	18.2	9.4	29.3
Benfuracarb	0.01	91.6	104.0	5.3	3.4	10.1	12.7	23.8
Bensulfuron-methyl	0.01	101.1	101.3	6.8	3.1	13.0	7.4	21.9
Bentazone	0.01	96.8	95.8	13.9	5.8	5.8	4.6	12.3
Bifenazate	0.01	101.4	105.4	9.7	3.5	7.1	4.1	14.9
Bifenthrin	0.01	102.5	103.6	4.6	3.8	9.4	5.6	19.6
Boscalid	0.01	91.4	107.0	11.0	4.5	4.8	9.6	16.7
Bromoxynil	0.01	106.0	101.5	7.7	5.7	9.2	8.8	16.0
Bromuconazole	0.01	85.7	101.7	9.8	8.1	11.3	4.9	21.3
Bupirimate	0.01	102.4	104.9	12.0	6.2	6.7	4.9	13.3
Buprofezin	0.01	93.0	103.0	18.1	4.7	4.6	13.3	21.2
Cadusafos	0.01	108.6	103.5	3.7	4.1	15.3	9.6	25.9
Carbaryl	0.01	92.1	103.6	15.6	7.5	9.5	9.0	19.8
Carbendazim	0.01	96.3	95.9	4.4	2.7	6.5	8.2	15.2
Carbofuran	0.01	105.6	105.5	9.9	2.5	18.5	6.6	28.3
Carbosulfan	0.01	88.7	104.0	13.6	5.9	14.8	10.8	26.8
Carboxin	0.01	82.2	104.7	12.5	3.6	12.8	8.3	22.7
Chlorantraniliprole	0.01	88.7	98.6	11.3	12.9	12.0	7.2	21.7
Chlorbromuron	0.01	106.3	104.7	13.6	7.4	17.5	8.7	28.9
Chlorfluazuron	0.01	96.9	101.1	7.3	4.7	8.3	3.8	14.3
Chloridazon	0.01	92.7	106.2	7.6	2.3	4.4	7.9	13.8
Chlorpyrifos	0.01	97.7	106.1	6.7	0.7	10.9	3.2	16.6
Chlorpyrifos-methyl	0.01	92.7	97.2	7.4	2.0	8.3	5.0	14.3
Chlorsulfuron	0.01	114.6	100.2	2.2	5.8	17.5	10.1	29.2
Clethodim	0.01	99.5	92.8	11.7	2.4	13.7	10.3	24.9
Clodinafop	0.01	78.4	107.0	8.5	1.3	7.3	3.0	12.0
Clofentezine	0.01	93.6	99.8	6.7	4.5	4.7	7.0	13.0
Clothianidin	0.01	108.4	102.1	7.7	1.8	17.9	3.2	26.4
Cyazofamid	0.01	102.3	99.8	8.1	7.3	12.9	4.7	20.7
Cycloate	0.01	111.3	99.6	2.8	4.8	11.1	2.6	16.6
Cyflumetofen	0.01	107.8	95.4	5.3	2.5	9.1	7.1	17.2
Cymoxanil	0.01	79.6	94.2	9.4	3.4	5.2	5.5	11.6

Cypermethrin	0.01	96.5	104.5	8.6	3.1	9.1	7.5	18.0
Cyproconazole	0.01	102.2	102.0	14.2	2.5	11.4	8.8	21.8
Cyprodinil	0.01	107.4	107.7	9.2	8.2	8.8	8.5	18.2
Dazomet	0.01	87.2	93.0	18.9	2.7	19.2	5.2	29.2
Deltamethrin	0.01	79.3	101.2	3.2	2.4	7.4	11.3	20.0
Demeton-S-methyl	0.01	109.0	101.2	7.2	6.0	6.0	7.3	14.8
Demeton-S-methyl-sulfone	0.01	96.1	101.2	11.2	4.2	10.8	5.5	18.4
Demeton-S-methyl-sulfoxide	0.01	91.2	104.0	16.3	4.1	10.9	9.8	21.8
Desmedipham	0.01	76.8	93.8	9.9	3.0	13.9	5.3	21.7
Diafenthiuron	0.01	101.9	101.6	8.4	3.6	14.3	14.8	24.7
Diazinon	0.01	113.6	103.8	4.2	4.4	18.4	2.9	27.8
Dichlofluanid	0.01	108.9	105.8	6.2	3.8	9.7	6.2	18.1
Dichlorvos	0.01	91.8	102.5	12.0	2.9	14.7	3.1	23.4
Dicrotophos	0.01	96.8	96.7	7.4	1.2	16.7	3.0	25.0
Diethofencarb	0.01	78.6	96.2	5.5	5.5	17.3	13.0	25.6
Difenoconazole	0.01	94.4	102.3	7.9	1.1	14.4	16.1	27.9
Diflubenzuron	0.01	81.4	104.2	7.1	3.6	13.4	6.4	30.4
Dimethenamid	0.01	102.4	103.3	7.2	5.0	4.5	7.4	10.3
Dimethoate	0.01	92.3	106.0	8.5	3.4	4.6	6.8	13.1
Dimethomorph	0.01	87.4	101.7	15.3	4.6	6.4	9.7	14.7
Dinocap	0.01	85.3	103.2	6.5	5.4	16.8	4.8	24.7
Dinotefuran	0.01	87.7	99.6	8.6	6.8	16.4	14.3	24.6
Dithianon	0.01	100.8	104.3	13.5	2.7	16.8	6.3	26.2
Diuron	0.01	91.9	96.2	11.0	2.2	8.4	3.4	14.8
Dodine	0.01	90.4	93.1	4.4	1.6	16.1	9.2	26.6
Epoxiconazole	0.01	105.9	99.0	6.7	4.3	15.3	11.3	27.4
Ethiofencarb	0.01	109.5	96.7	3.5	4.0	19.6	12.9	33.4
Ethofumesate	0.01	109.2	97.9	5.1	5.0	19.8	6.6	30.0
Etofenprox	0.01	107.9	100.6	3.5	1.6	10.3	14.3	25.2
Etoxazole	0.01	97.9	100.7	10.7	5.8	6.3	16.2	25.3
Famoxadone	0.01	111.1	99.7	8.6	3.4	19.7	11.7	33.0
Fenamidone	0.01	112.1	90.5	14.0	2.3	17.6	4.4	26.4
Fenarimol	0.01	104.3	105.4	6.1	3.8	13.8	12.8	27.0
Fenazaquin	0.01	110.4	100.9	2.7	3.9	5.5	18.0	26.9
Fenbuconazole	0.01	96.6	101.2	14.5	4.2	4.5	11.0	17.8
Fenhexamid	0.01	99.9	100.3	6.3	6.9	8.9	15.0	25.3
Fenoxaprop-P-ethyl	0.01	93.1	110.5	7.2	3.6	11.8	10.5	22.9
Fenoxycarb	0.01	98.8	102.4	3.4	5.5	7.2	2.3	11.5
Fenpropathrin	0.01	108.1	105.2	5.3	3.3	11.1	4.4	17.3
Fenpyroximate	0.01	94.4	91.8	11.3	2.2	10.7	5.0	18.3
Fenthion	0.01	88.8	105.3	13.3	4.5	8.7	4.0	14.6
Flonicamid	0.01	102.7	103.1	4.2	3.9	7.4	4.7	13.8
Fluazinam	0.01	95.1	101.8	7.6	2.5	3.7	7.7	7.0
Flubendiamide	0.01	86.3	102.4	7.5	1.7	16.1	3.7	24.0
Fludioxonil	0.01	110.5	100.3	1.6	3.9	18.9	3.5	27.5
Flufenoxuron	0.01	99.5	103.0	6.4	4.2	7.7	6.1	15.6
Fluopicolide	0.01	108.8	107.4	11.2	4.4	8.0	2.5	13.4
Fluopyram	0.01	89.4	97.1	10.1	4.7	4.8	6.8	12.9
Flupyradifurone	0.01	82.9	105.3	12.6	1.3	13.1	3.9	19.8
Flutriafol	0.01	106.0	97.3	8.5	4.3	7.9	7.7	16.6
Fluxapyroxad	0.01	103.2	97.1	5.7	8.5	18.9	8.1	30.1
Fonofos	0.01	83.1	95.2	11.8	6.6	11.7	3.7	18.2
Formetanate	0.01	106.0	104.7	12.0	2.1	5.3	11.3	18.8
Fosthiazate	0.01	105.3	98.8	6.0	3.9	10.1	4.2	16.0
Furathiocarb	0.01	92.3	104.0	16.3	3.7	18.8	9.2	31.3

Haloxyfop	0.01	104.6	98.9	8.0	7.0	15.1	3.6	22.6
Haloxyfop-P-methyl	0.01	82.0	108.7	7.2	4.3	16.3	9.3	27.4
Heptenophos	0.01	83.4	110.9	7.0	3.8	8.5	6.0	15.3
Hexaconazole	0.01	102.5	101.3	11.4	4.2	17.8	13.4	32.0
Hexaflumuron	0.01	108.2	104.8	6.1	3.2	10.3	7.5	18.5
Hexythiazox	0.01	92.1	103.5	5.6	6.0	7.5	18.3	28.6
Imazalil	0.01	110.2	100.7	3.9	6.1	5.5	5.9	13.8
Imidacloprid	0.01	104.8	98.9	9.0	6.4	10.9	5.0	18.2
Indoxacarb	0.01	108.2	106.0	5.4	4.7	6.2	7.2	15.1
Ioxynil	0.01	101.4	104.6	5.3	5.3	11.2	5.8	17.7
Iprodione	0.01	93.8	104.8	13.1	7.0	12.1	6.2	21.4
Iprovalicarb	0.01	86.3	101.7	12.4	4.7	3.5	10.3	11.4
Isoprocarb	0.01	82.3	102.0	9.9	6.5	7.0	7.4	14.6
Isopyrazam	0.01	105.5	83.4	8.6	2.4	17.4	2.7	27.0
Kresoxim-methyl	0.01	106.8	101.2	7.7	6.5	10.6	8.3	20.0
Lambda-cyhalothrin	0.01	93.9	101.9	12.6	2.6	10.6	4.0	17.3
Lenacil	0.01	99.8	103.1	15.5	7.8	11.3	16.8	29.9
Linuron	0.01	89.2	104.5	18.8	6.3	3.6	3.9	10.8
Lufenuron	0.01	78.6	94.6	8.5	5.1	5.1	8.5	15.6
Malaoxon	0.01	87.8	98.0	8.1	8.9	3.7	13.4	20.4
Malathion	0.01	88.5	99.2	12.9	3.6	12.3	13.0	26.5
Mandipropamid	0.01	101.0	105.7	6.3	7.2	12.1	12.0	25.4
Mecarbam	0.01	107.8	102.9	8.7	1.1	15.4	13.7	29.6
Metalaxyl	0.01	95.0	99.3	3.8	5.5	3.8	11.6	17.6
Metamitron	0.01	96.1	103.9	10.4	5.9	9.8	9.7	20.1
Methacrifos	0.01	89.6	98.5	15.6	7.1	13.5	9.3	24.8
Methamidophos	0.01	87.9	101.4	9.9	6.2	5.4	4.6	11.3
Methiocarb	0.01	101.0	101.6	4.0	6.2	17.8	14.8	33.1
Methomyl	0.01	91.3	104.3	18.3	2.6	9.7	14.3	26.5
Metolachlor	0.01	110.7	100.3	7.9	5.8	17.0	8.0	27.0
Metribuzin	0.01	108.5	102.4	6.8	4.7	14.3	11.4	26.3
Mevinphos	0.01	103.1	106.5	9.9	2.3	6.6	8.3	15.8
Monocrotophos	0.01	91.6	94.7	3.1	2.8	11.6	7.2	19.9
Monolinuron	0.01	104.0	98.1	10.7	5.8	12.9	10.0	23.8
Myclobutanil	0.01	110.2	108.8	11.0	3.1	14.5	15.9	31.5
Nicosulfuron	0.01	79.5	95.5	10.4	2.0	19.6	3.7	28.8
Nuarimol	0.01	99.3	106.3	4.8	7.2	5.9	4.3	12.0
Omethoate	0.01	86.1	102.9	11.1	3.8	6.0	4.1	12.2
Oxadixyl	0.01	101.4	105.7	6.1	4.1	15.9	8.5	25.7
Oxamyl	0.01	109.4	105.3	10.9	6.0	7.0	16.4	25.8
Oxyfluorfen	0.01	110.8	75.7	7.1	4.3	3.9	6.5	17.0
Paraoxon-ethyl	0.01	101.4	100.7	10.1	5.8	7.3	3.4	13.7
Penconazole	0.01	107.7	97.8	10.5	5.1	14.6	11.4	26.9
Pendimethalin	0.01	100.9	106.2	11.7	3.8	8.8	13.3	23.2
Phenmedipham	0.01	79.2	109.6	7.1	3.2	7.1	3.9	12.3
Phenthoate	0.01	112.0	100.4	4.7	8.0	4.5	11.3	18.0
Phorate	0.01	94.4	102.6	12.6	6.9	6.6	4.9	20.1
Phosalone	0.01	102.0	104.9	4.8	6.2	14.5	5.7	22.5
Phosmet	0.01	116.9	107.3	2.7	3.9	19.7	10.1	31.6
Phosphamidon	0.01	107.9	106.7	5.3	4.3	7.3	12.2	20.6
Pirimicarb	0.01	105.1	105.7	5.8	7.8	5.1	6.6	12.6
Pirimiphos-ethyl	0.01	96.1	91.3	16.1	2.3	13.5	4.6	21.2
Pirimiphos-methyl	0.01	95.2	104.7	5.1	9.2	7.9	10.7	19.4
Prochloraz	0.01	104.9	106.2	3.3	7.0	15.6	8.9	26.0
Profenofos	0.01	104.5	105.3	3.1	6.1	8.7	17.0	27.3

Profoxydim	0.01	100.8	89.1	11.9	1.9	17.1	8.0	27.9
Promecarb	0.01	101.3	94.6	8.5	4.4	11.6	14.4	33.7
Prometryn	0.01	106.1	109.7	7.8	2.4	9.2	10.4	20.2
Propamocarb	0.01	99.7	109.7	4.1	2.4	4.9	9.5	15.6
Propaquizafop	0.01	100.8	99.6	11.2	2.2	10.7	7.9	19.4
Propargite	0.01	114.6	104.1	2.6	6.6	12.1	10.6	23.3
Propazine	0.01	87.8	102.5	17.7	6.8	3.3	7.3	14.0
Propiconazole	0.01	109.0	105.3	6.0	3.7	7.8	13.2	22.3
Propoxur	0.01	109.4	108.2	2.0	4.3	11.5	4.3	18.1
Propyzamide	0.01	103.4	104.6	12.9	6.0	3.5	7.9	15.0
Prosulfuron	0.01	82.3	108.8	13.3	5.9	12.3	7.4	21.3
Prothiofos	0.01	111.4	110.0	3.8	1.6	18.2	13.2	31.9
Pymetrozine	0.01	107.3	106.8	5.4	1.9	17.8	6.8	27.4
Pyraclostrobin	0.01	88.2	105.6	7.0	1.8	9.2	15.3	26.0
Pyrazophos	0.01	113.3	104.2	3.8	2.8	17.3	11.5	29.6
Pyridaben	0.01	83.5	103.2	2.3	3.8	16.2	11.1	25.1
Pyridaphenthion	0.01	105.4	102.4	3.9	4.9	13.6	11.2	25.2
Pyridate	0.01	89.6	93.8	15.6	1.9	17.8	7.1	28.2
Pyrimethanil	0.01	102.3	92.9	8.3	2.1	15.3	5.2	24.9
Pyriproxyfen	0.01	107.7	105.9	5.4	5.9	15.6	11.0	27.2
Rimsulfuron	0.01	107.8	103.1	6.5	5.1	8.2	9.5	18.5
Sethoxydim	0.01	107.0	88.4	5.8	2.9	11.6	5.7	18.8
Simazine	0.01	91.3	108.2	17.7	4.9	5.0	5.1	13.5
Spinetoram	0.01	91.1	104.6	9.0	1.6	15.9	6.4	23.1
Spinosyn A	0.01	106.4	100.7	5.6	5.4	6.0	7.1	21.1
Spinosyn D	0.01	102.3	98.6	5.2	5.1	5.9	6.6	19.8
Spirodiclofen	0.01	95.9	103.4	3.2	1.4	14.8	5.0	23.4
Spiromesifen	0.01	79.2	94.2	13.9	3.1	11.8	6.8	21.8
Spirotetramat	0.01	103.9	112.2	3.2	2.4	14.3	10.6	23.0
Spirotetramat-enol	0.01	103.7	100.0	8.6	5.2	16.7	7.1	26.8
Spiroxamine	0.01	104.4	104.6	6.1	4.9	6.0	8.2	14.8
Sulfoxaflor	0.01	109.7	105.7	5.3	2.0	18.6	13.9	33.1
Tau-fluvalinate	0.01	100.0	99.3	5.9	3.7	16.2	4.1	25.0
Tebuconazole	0.01	84.4	89.3	10.8	2.1	11.8	12.0	24.4
Tebufenozide	0.01	105.8	106.5	7.2	9.1	13.2	14.6	28.7
Tebufenpyrad	0.01	80.3	102.8	8.5	1.6	6.2	10.7	17.9
Teflubenzuron	0.01	104.4	92.1	5.1	4.2	8.3	7.6	16.5
Tepraloxydim	0.01	96.3	100.6	9.7	5.6	11.8	9.3	22.1
Terbufos	0.01	111.2	105.8	3.5	5.7	19.5	12.5	33.0
Terbuthylazine	0.01	89.8	108.2	10.3	3.7	6.6	9.0	16.8
Terbuthylazine-desethyl	0.01	111.5	105.8	5.8	6.8	16.5	8.8	27.2
Terbutryn	0.01	112.7	109.9	2.9	4.9	13.1	11.0	24.4
Tetraconazole	0.01	101.8	104.3	10.4	7.5	13.8	12.4	27.3
Thiabendazole	0.01	113.2	108.0	7.3	1.9	9.8	9.4	15.4
Thiacloprid	0.01	110.5	105.7	4.4	6.0	12.5	9.7	20.7
Thiamethoxam	0.01	102.5	109.1	7.1	6.1	16.2	5.2	26.9
Thifensulfuron-methyl	0.01	105.8	91.5	10.3	2.3	12.5	4.8	23.2
Thiobencarb	0.01	104.7	98.4	9.7	2.5	9.0	10.9	16.1
Thiodicarb	0.01	109.9	102.1	6.6	5.4	18.9	10.6	31.1
Thiophanate-methyl	0.01	86.6	110.1	9.9	3.8	6.7	7.1	13.0
Tolyfluanid	0.01	101.7	106.3	15.3	5.0	12.8	14.2	27.8
Tralkoxydim	0.01	107.0	89.9	7.8	2.2	9.1	5.5	15.5
Triadimefon	0.01	103.6	106.4	12.4	4.6	10.2	16.3	18.4
Triadimenol	0.01	106.0	103.1	9.1	4.9	19.2	5.4	28.1
Triallate	0.01	88.9	96.1	13.5	2.6	7.9	4.8	13.7

Triasulfuron	0.01	104.4	102.7	12.8	5.0	10.9	4.4	18.6
Trichlorfon	0.01	95.6	103.8	12.1	5.8	7.2	12.9	14.4
Trifloxystrobin	0.01	110.2	105.5	1.4	5.7	18.6	7.8	31.0
Triflumizole	0.01	107.4	105.8	8.2	5.8	14.6	5.4	22.6
Triflumuron	0.01	102.1	105.9	8.7	5.4	15.2	8.7	23.0
Triticonazole	0.01	105.3	110.9	4.8	5.2	15.1	5.4	25.1
Valifenalate	0.01	96.8	95.8	5.6	2.0	14.1	2.3	21.4
Vamidothion	0.01	89.7	104.3	16.2	3.3	10.5	4.8	17.9
Zoxamide	0.01	102.1	104.6	16.8	7.7	6.7	12.6	21.5

Table S4. Definition and individual scores of indices for the pesticide residual risk ranking.

**Categories**	**Definition**	**Score**	**Definition**	**Score**	**Definition**	**Score**	**Definition**	**Score**
A: Toxicity (LD_{50})	Low toxicity	2	Moderate toxicity	3	Highly toxicity	4	Rank poison	5
B: Toxic effect (ADI)	＞0.01	0	0.0001–0.01	1	0.000001–0.0001	2	＜0.000001	3
C: Proportion of diet (%)	＜2.5	0	2.5–20	1	20–50	2	50–100	3
D: Frequency of pesticide (%)	＜2.5	0	2.5–20	1	20–50	2	50–100	3
E: High-exposure crowd	nothing	0	few	1	mainly	2	indetermin acy	3
F: Residual level	ND	1	＜1MRL	2	1–10 MRL	3	≥ 10 MRL	4

Table S5. Assigned scores for indices A-F used in the pesticide residue risk scoring system.

**Pesticide**	**Toxicity (LD_{50}) A**	**Toxic effect (ADI) B**	**Proportion of diet C**			**Frequency of pesticide D**			**High-exposure crowd E**	**Residual level (mg/kg)* F**		
			**Apple**	**Pear**	**Quince**	**Apple**	**Pear**	**Quince**		**Apple**	**Pear**	**Quince**
Acetamiprid	3	1	1	0	–	0	0	–	3	1.45	1.42	–
Azoxystrobin	2	0	–	0	–	–	0	–	3	–	0.98	–
Boscalid	2	2	1	0	0	0	0	0	3	1.18	1.36	1.10
Chlorantraniliprole	2	2	1	0	0	0	0	0	3	1.13	1.06	1.12
Cyflumetofen	2	0	1	–	–	0	–	–	3	1.07	–	–
Cyprodinil	2	1	–	0	–	–	0	–	3	–	1.04	–
Deltamethrin	3	1	–	–	0	–	–	0	3	–	–	1.06
Difenoconazole	3	0	1	0	–	0	1	–	3	1.02	1.48	–
Dithianon	3	1	–	0	–	–	1	–	3	–	1.04	–
Dodine	3	0	–	–	0	–	–	0	3	–	–	1.06
Etofenprox	2	0	–	0	–	–	0	–	3	–	1.08	
Fluopyram	2	0	–	–	0	–	–	0	3	–	–	1.04
Flupyradifurone	3	0	–	0	–	–	0	–	3	–	1.02	–
Fluxapyroxad	2	1	1	–	–	0	–	–	3	1.05	–	–
Lambda-cyhalothrin	3	1	–	–	0	–	–	0	3	–	–	1.20
Malathion	2	0	–	0	–	–	0	–	3	–	1.08	
Pyraclostrobin	2	0	–	0	0	–	0	0	3	–	1.06	1.10
Pyridaben	3	1	1	–	–	0	–	–	3	1.18	–	–
Pyrimethanil	2	0	1	0	–	0	0	–	3	1.09	1.44	–
Sulfoxaflor	2	0	1	–	–	0	–	–	3	1.05	–	–
Tebuconazole	3	0	1	0	0	0	0	0	3	1.11	1.14	1.04
Tebufenpyrad	3	1	1	0	–	0	0	–	3	1.07	1.06	–
